# Octakis(dodecyl)phthalocyanines: Influence of Peripheral versus Non-Peripheral Substitution on Synthetic Routes, Spectroscopy and Electrochemical Behaviour

**DOI:** 10.3390/molecules27051529

**Published:** 2022-02-24

**Authors:** Glendin Swart, Eleanor Fourie, Jannie C. Swarts

**Affiliations:** Department of Chemistry, University of the Free State, Bloemfontein 9300, South Africa; 2015271865@ufs4life.ac.za (G.S.); swartsjc@ufs.ac.za (J.C.S.)

**Keywords:** phthalocyanine, metal-free, zinc, magnesium, UV–vis, FTIR, NMR, XPS, cyclic voltammetry

## Abstract

Non-peripherally octakis-substituted phthalocyanines (**np**Pc’s), MPc(C_12_H_25_)_8_ with M = 2H (**3**) or Zn (**4**), as well as peripherally octakis-substituted phthalocyanines (**p**Pc’s) with M = Zn (**6**), Mg (**7**) and 2H (**8**), were synthesized by cyclotetramerization of 3,6- (**2**) or 4,5-bis(dodecyl)phthalonitrile (**5**), template cyclotetramerization of precursor phthalonitriles in the presence of Zn or Mg, metal insertion into metal-free phthalocyanines, and removal of Mg or Zn from the phthalocyaninato coordination cavity. The more effective synthetic route towards **p**Pc **8** was demetalation of **7**. **np**Pc’s were more soluble than **p**Pc’s. The Q-band λ_max_ of **np**Pc’s was red-shifted with ca. 18 nm, compared to that of **p**Pc’s. X-ray photoelectron spectroscopy (XPS) differentiated between N–H, N_meso_ and N_core_ nitrogen atoms for metal-free phthalocyanines. Binding energies were ca. 399.6, 398.2 and 397.7 eV respectively. X-ray photoelectron spectroscopy (XPS) also showed zinc phthalocyanines **4** and **6** have four equivalent N_meso_ and four equivalent N–Zn core nitrogens. In contrast, the Mg phthalocyanine **7** has two sets of core N atoms. One set involves two N_core_ atoms strongly coordinated to Mg, while the other encompasses the two remaining N_core_ atoms that are weakly associated with Mg. **p**Pc’s **6**, **7**, and **8** have cyclic voltammetry features consistent with dimerization to form [Pc][Pc^+^] intermediates upon oxidation but **np**Pc’s **3** and **4** do not. Metalation of metal-free **p**Pc’s and **np**Pc’s shifted all redox potentials to lower values.

## 1. Introduction

**Figure 1:**
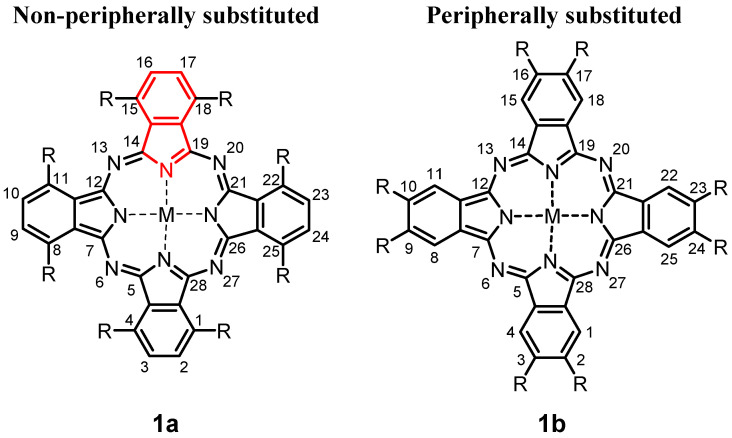
Structure of non-peripherally (**1a**) and peripherally (**1b**) octakis-substituted phthalocyanines. R = any suitable substituent. The central coordinated atom, M, can be one of at least 70 different elements of the periodic table.

Phthalocyanines represent a class of chromophores, which consist of four isoindole subunits (red fragment highlighted in Figure 1), tethered together by aza-bridges, resulting in an 18 π-electron aromatic configuration [1]. Cyclization of isoindole fragments, in this manner, produces phthalocyanines, bearing a cavity that can accommodate ca. 70 elements of the periodic table.

Protons situated on peripheral positions (positions 2, 3, 9, 10, 16, 17, 23, and 24 of structure **1a** in Figure 1) are simultaneously *meta* and *para* relative to the pyrrolic fragment of the phthalocyanine. The properties of these protons differ from those of non-peripheral phthalocyanine protons (positions 1, 4, 8, 11, 15, 18, 22, and 25 of phthalocyanine **1b** in Figure 1), and this positioning is always *ortho* relative to the pyrrolic fragment of the phthalocyanine. The latter positions are more protected (or sterically hindered) than the former in the presence of bulky R groups. As such, peripheral and non-peripheral protons in **1a** and **1b** exist in chemically inequivalent environments. They are aromatic protons because of the aromaticity of the 18 π-electron phthalocyanine macrocycle.

The purpose of bulky periphery substituents R (Figure 1) or large axial ligands coordinated to metal ions within the phthalocyaninato core (M in Figure 1) is to mitigate the aggregation propensity of phthalocyanines, so that chemical and physical properties of the monomeric phthalocyaninato species can be efficiently harvested for industrial or technological applications. 

Appropriate augmentation to achieve suitable phthalocyanine derivative solubility (or modulate any other physical or chemical property) can be brought about by substitution on the periphery of the annulated benzene rings, complexation with a metal centre with axial ligands to the phthalocyanine core, or by changing the nature of the conjugated π-system [1]. Peripheral and/or non-peripheral substitution is achieved by either aromatic electrophilic substitution or cycloaddition to a preformed unsubstituted phthalocyanine or through the cyclotetramerization of the appropriately substituted precursor [2]. The latter strategy provides more control over the number of substituents and phthalocyanine isomers formed during the reaction [2,3]. Metal phthalocyanines can be prepared through a metal-template directed cyclotetramerization between a phthalocyanine precursor, such as phthalonitriles, and corresponding metal salt [4]. Ince and co-workers demonstrated this with the synthesis of peripherally substituted octakis(dodecyl)phthalocyaninatozinc(II) [5]. Metalation can also be performed on a pre-existing metal-free phthalocyanine, through refluxing in the presence of a metal salt [6]. Metal carboxylates are preferred over metal halides as metal sources.

The delocalised, conjugated π-system of a phthalocyanine derivative can undergo excitation through photon interactions, resulting in intense Q-band absorption maxima in the range of ca. 620–700 nm (two Q-band components, Q_x_ and Q_y_, are observed for metal-free phthalocyanines) and a lower intensity Soret band absorption maximum in the range of 320–420 nm. 

Electric field excitation causes metal-free phthalocyanines, or those containing a redox-silent metallic centre, to normally exhibit a total of six ring-based, one-electron redox processes. Two of these are oxidations, while the remainder are reductions [7]. Electrochemical analysis of suitably soluble phthalocyanines, within the solvent window of dichloromethane, often reveal four of the six ring-based redox couples [8], although 17 of the possible 18 one-electron transfer processes of a cadmium triple decker phthalocyanine, Cd_2_Pc_3_, were observed in the solvent THF [9]. Phthalocyanines bearing four non-peripheral 2-butyloctyloxy sidechains have been shown to form condensed phases and exhibit liquid-crystal behaviour [10].

Substitution on the periphery and metalation have been shown to change solution phase aggregation [11,12], cause discotic mesophase behaviour [13], alter electrochemical properties [14], and shift the wavelength for Q-band absorption maxima [15]. Using a combination of these augmentations allows for the unique physical and chemical properties of phthalocyanines to be fine-tuned. This process of fine-tuning allows phthalocyanines to be utilised in areas such as photodynamic cancer therapy (PDT) [16], bulk heterojunction organic solar cells [17], oxygen reduction reaction catalysis [18], nonlinear optics [19], and liquid crystal display technology [20].

**Scheme 1:**
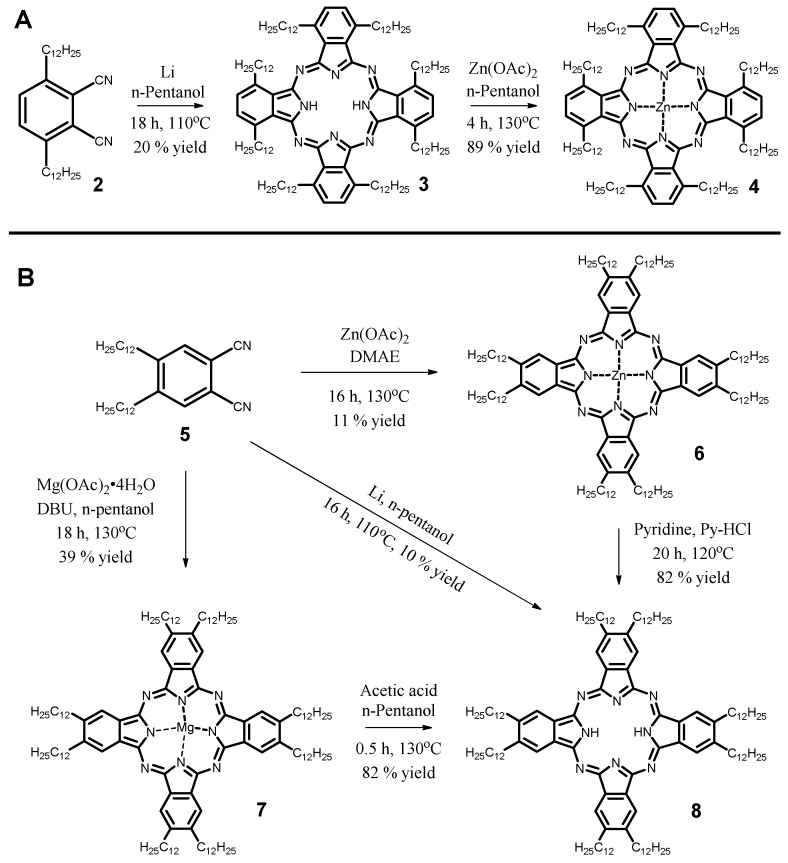
(**Top**, **A**) Synthesis of non-peripherally substituted phthalocyanines. (**Bottom**, **B**) Synthesis of peripherally substituted phthalocyanines. Consistent with XPS results, described below, N-binding to Mg in **7** is not shown as four equivalent symmetric bonds, but rather as two inequivalent sets of bonding interactions. DMAE = dimethylaminoethanol, DBU = 1.8-diazabicyclo[5,4,0]undec-7-ene, OAc = acetate, Py = pyridine.

The synthesis, UV–vis spectrometry, and ^1^H NMR results for non-peripherally substituted octakis(dodecyl)phthalocyanine, **3**, and its zinc-coordinated derivative **4**, Scheme 1, have been reported [3]. Cyclic voltammetry of **3** in dichloromethane (DCM) at 25 °C and that of **4** in dichloroethane (DCE) at 70 °C were presented. Tetrabutylammonium hexafluorophosphate ([N(^n^Bu)_4_][PF_6_]) was utilised as a supporting electrolyte. A limitation of this electrochemical study was that potentials were reported versus an Ag/Ag^+^ reference because, when ferrocene was added as an internal reference, it was found to overlap with the first ring-based oxidation. In addition, PF_6_^−^ anions (from the supporting electrolyte used) are known to form ion pairs with cationic (oxidised) species. Phthalocyanine **6**, peripherally substituted octa(dodecyl)phthalocyananinatozinc(II), Scheme 1, was previously isolated from a phthalocyanine mixture produced from a statistical condensation [5]. The UV–vis spectroscopy, ^1^H NMR and mesophase behaviour were reported. The metal-free derivative of this complex was not synthesised, and neither were any electrochemical studies performed.

With this background, in the present work, the synthesis of **3**, **4**, **6**, and the new metal-free and magnesium derivatives of **6** phthalocyanines **8** and **7**, respectively, is presented. Special focus has been placed on the synthesis of **8**, Scheme 1. Three different synthetic routes were employed. Cyclic voltammetric results of all phthalocyanines were obtained here in the common solvent THF and supporting electrolyte [N(^n^Bu_4_)][B(C_6_F_6_)_4_]. This allows direct comparison and identification of electrochemical differences, as a result of different substitution patterns, peripherally vs. non-peripherally. The use of the supporting electrolyte [N(^n^Bu_4_)][B(C_6_F_6_)_4_] is beneficial, as the B(C_6_F_6_)_4_^−^ anion is known to be inert to ion pair formation with cationic species [21]. All formal reduction potentials are here referenced vs. FcH/FcH^+^, and the internal reference, decamethylferrocene, has been chosen, which does not overlap/interfere with any phthalocyanine redox properties. Together with ^1^H NMR, UV–vis, and X-ray photoelectron spectroscopy (XPS), we highlight the influence peripheral versus non-peripheral substitution have on the spectroscopy and electrochemical properties of octakis(dodecyl)phthalocyanines.

## 2. Results and Discussion

### 2.1. Synthesis

The precursor phthalonitrile **2**, 3,6-bis(dodecyl)phthalonitrile was obtained from thiophene using a four-step reaction sequence, as is described previously [3]. Thiophene was first lithiated with n-BuLi to form 2,5-dilithiumthiophene, which was treated in situ with 1-bromododecane to replace both lithium atoms with dodecyl substituents. Dimethyldioxirane was then used to oxidise 2,5-bis(dodecyl)thiophene to the corresponding thiophene-1,1-dioxide (or sulfone). The five-membered sulfone ring was expanded to a six-membered benzene by means of Diels–Alder condensation with fumaronitrile to liberate, after SO_2_ elimination, phthalonitrile **2**. Both the reaction sequence (Scheme S1) and experimental details of the synthesis of **2** may be found in the Supporting Information.

Precursor **5**, 4,5-bis(dodecyl)phthalonitrile, required no ring expansion procedures. It was synthesised from phthalimide using a modified five-step reaction sequence, as described in literature [22,23]. Electrophilic aromatic iodination of phthalimide, using elemental iodine and fuming sulfuric acid, was followed by imide ring opening in aqueous ammonia to yield 4,5-diiodobenzene-1,2-dicarboxamide. Subsequent dehydration with either trifluoroacetic anhydride or phosphoryl chloride forms 4,5-diiodophthalonitrile, the iodine atoms of which were replaced with dodecyne substituents in a follow-up modified Sonogashira coupling with 1-dodecyne. To obtain phthalonitrile **5**, the alkyne groups were then converted to an aliphatic functionality, by means of catalytic hydrogenation, in the presence of palladium on carbon. Modified experimental conditions, as well as a detailed reaction sequence (Scheme S2), may be found in the Supporting Information.

Metal-free non-peripherally substituted phthalocyanine **3** was synthesised under argon through cyclotetramerization of **2**, using lithium pentoxide as nucleophilic initiator (Scheme 1). Lithium pentoxide was generated in situ through the addition of metallic lithium to warm pentanol. After acidic workup and column chromatography **3** was obtained in 20% yield as an air-stable dark green solid. Zinc phthalocyanine **4** was obtained as a blue solid, in 89% yield, after workup by refluxing **3** under dry and air-free conditions in n-pentanol, in the presence of anhydrous zinc acetate. Both **3** and **4** should be stored in a dark environment, as photodegradation of these compounds was observed on TLC plates, which were exposed to laboratory lighting for short periods of time. 

The peripherally substituted zinc phthalocyanine **6** was obtained by refluxing 4,5-bis(dodecyl)phthalonitrile **5** and anhydrous zinc acetate in dimethylaminoethanol, under dry and air-free conditions. This contrasts Ince’s method [5], where **6** was obtained as a side product from a statistical condensation between **5** and a differently substituted phthalonitrile.

However, no metal-free derivative of **6**, i.e., phthalocyanine **8** in Scheme 1, is known. Searching for a method to synthesise the metal-free peripherally substituted octakis(dodecyl)phthalocyanine **8**, we noted that Cammidge and co-workers reacted two equivalents of methyl magnesium bromide with 4,5-bis(2′-ethylhexyl)phthalonitrile. This yielded, in their hands, both magnesium phthalocyanine and tetrabenzotriazaporphyrin, with a ratio of 4:5 [24]. The central magnesium metal can then easily be removed to generate the metal-free species. Attempts of obtaining the magnesium phthalocyanine, **7**, through this method failed, even if the number of equivalents of methyl magnesium bromide was lowered to 1. ^1^H NMR of the isolated green residue indicated that only the tetrabenzotriazaporphyrin had formed. Duplication of the synthetic procedure of **6**, but by substituting anhydrous zinc acetate with magnesium acetate tetrahydrate, also failed to generate magnesium phthalocyanine **7**. Only a dark yellow, unidentified oil was obtained after workup. However, as shown in Scheme 1, addition of the strong, hindered base, DBU (1.8-diazabicyclo[5,4,0]undec-7-ene), into the reaction mixture allowed for the generation of the magnesium phthalocyanine, **7**, in 39% yield after workup. The lower reactivity of Mg(OAc)_2_.4H_2_O, compared to Zn(OAc)_2_, is likely attributed to water of crystallization, which impedes the ability of the acetate ion to act as a cyclisation initiator.

Removal of the magnesium central metal from **7**, with acetic acid at moderate temperatures (130 °C), generated the metal-free phthalocyanine **8**, within 0.5 h in high (82%) yield, Scheme 1. Removal of the zinc central metal from **6** required more forcing conditions. Acetic acid was replaced with pyridine-HCl, and the reaction time increased to 20 h at 120 °C. Yields of **8** were, however, still high (82%). The direct approach to obtain **8** by refluxing **5** for 16 h in pentanol in the presence of lithium pentoxide, similar to the synthesis of **3**, only resulted in **8** in 10% yield. A key aspect here is that **8** cannot be chromatographed. Poor solubility causes **8** to sit on the top of the column, as if it is an impurity, even if pure THF is used as eluent. Once this was realised, the direct route became viable because **8** can be purified by crystallisation from *warm* THF, see experimental section.

To understand why the non-peripherally substituted metal-free phthalocyanine **3** is soluble, even in hexane, but the peripherally substituted metal-free phthalocyanine **8** is only weakly soluble in warm THF, one needs to look at the crystal structures of equivalent compounds. Cook solved the structure of a compound similar to **3**; the substituent was not -C_12_H_25_ but -C_6_H_13_ [25]. While it is known that n-alkyl chains tend to lie in straight lines in the solid state, this is not possible for all substituents in non-peripheral positions. Here, adjacent alkyl groups will collide with each other if they are orientated linearly away from the phthalocyanine macrocycle. Cook found that four of the eight alkyl substituents are protruding away from the macrocycle and lie in the same plane as the macrocycle. Two more are approximately in the plane of the macrocycle. This is caused by rotation about C–C bonds to allow some of the side chain’s C-atoms to be above the macrocycle plane, with some others below it. The last two side chains rotated in such a way that it points perpendicularly away from the macrocyclic plane; one points up and the other down. These two side chains act as spacers that push adjacent macrocycles apart. The distance between macrocyclic planes is 8.5 Å. This implies a very weak core:core interaction and explains the good solubility of non-peripherally substituted compounds, such as **3**. It will be easy for solvent molecules to diffuse between macrocycle cores, break any of the weak interactions that exist between them, and ultimately dissolve the phthalocyanine. In contrast, the alkyl substituents in peripherally substituted phthalocyanines, such as **8**, do not have to rotate in a way that they end up perpendicular to the macrocyclic plane. Phthalocyanine macrocycles of octakis peripherally substituted compounds were, therefore, found to be much closer to each other, only 3.4–3.6 Å [5,26]. Core:core interactions are much stronger, and solvent molecules do not break these interactions easily in the absence of heat (**8** was only soluble in THF at 60 °C).

We conclude that the most effective way of obtaining peripherally substituted metal-free phthalocyanine **8** is by first synthesizing the magnesium derivative **7**, followed by demetalation.

### 2.2. ^1^H NMR

For metalated phthalocyanines, the non-peripheral hydrogens of **6** and **7** have ^1^H NMR resonances at 9.23 and 9.25 ppm, respectively, in THF-*d*_8_, while the ^1^H NMR resonance of peripheral hydrogens of **4** is at 7.88 ppm, also in THF-*d*_8_. This drift of ca. 1.36 ppm is the result of the structural differences between the peripheral and non-peripheral complex. The non-peripheral aromatic hydrogens of phthalocyanines **6** and **7** are sterically hindered by the peripheral dodecyl groups (Scheme 1). The peripheral hydrogens of **4** are more exposed (“naked”) and less deshielded.

The influence of metalation on the resonance position of aromatic protons is demonstrated when one compares the aromatic proton resonances of **4**, **6**, and **7** with the corresponding resonances of **3** and **8**. In the peripherally substituted compound series **6**, **7**, and **8**, metalation caused a deshielding (movement of resonances to larger ppm values) of 0.19 (Zn) and 0.21 ppm (Mg) in THF-*d*_8_, while for non-peripherally substituted compounds, metalation had almost no effect. The aromatic proton resonance of **3** and **4** were both observed within the range of 7.87–7.88 ppm, despite being recorded in different solvents (CDCl_3_ for **3**; THF-*d*_8_ for **4**).

The difference in dodecyl substitution pattern also had an effect on the ^1^H NMR resonance position of the “benzylic” methylene protons (i.e., protons of the CH_2_ groups bound directly to the phthalocyanine core). Changing from a non-peripheral to a peripheral substitution pattern shifted the benzylic CH_2_ resonance of metal-free phthalocyanine **8** upfield by ca. 4.45 − 3.24 = 1.21 ppm, compared to the same resonance of **3**, while this resonance of the metalated peripherally substituted derivatives **6** and **7** experienced an upfield shift of ca. 4.62 − 3.26 = 1.36 ppm, compared to that of **4**. Zinc insertion into the phthalocyanine core had only a small upfield influence on the benzylic CH_2_ resonance position of the non-peripherally substituted compound series **3** and **4** (4.62 ppm − 4.45 ppm = 0.17 ppm). In the peripherally substituted compound series, **6**, **7**, and **8**, metalation had no effect on the benzylic CH_2_ resonance. For these three complexes, the benzylic proton resonance position was virtually unaltered at 3.24–3.26 ppm. Other aliphatic resonance positions were at expected positions and are documented in the experimental section. ^1^H NMR spectra may be found in the Supporting Information (Figure S9–S13).

### 2.3. FTIR–ATR and UV–Vis Spectroscopy

Successful cyclotetramerization of phthalonitriles **2** and **5** into phthalocyanines **3**, **6**, **7**, and **8** is reflected by the absence of the CN stretching vibration at 2229 cm^−1^ in the phthalocyanine Fourier transform infra-red attenuated total reflectance (FTIR–ATR) spectra, Supporting Information, Figure S14. FTIR–ATR spectra of the metal-free phthalocyanines **3** and **8** differ from those of the metalated phthalocyanines **4**, **6**, and **7** in that they show an additional stretching vibration at 3294 and 3358 cm^−1^, respectively. These vibrations are characteristic of the N–H bond within the phthalocyanine core.

**Figure 2:**
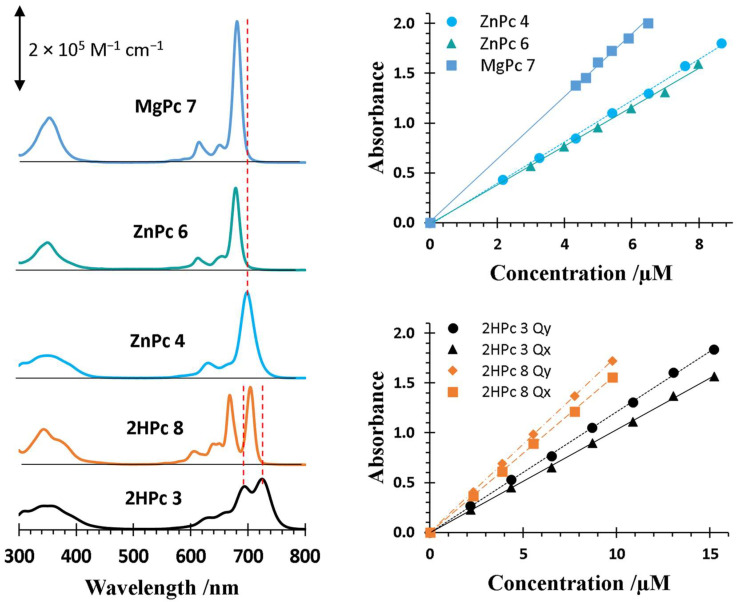
(**Left**) UV–vis spectra of phthalocyanines **3**, **4**, **6**, **7**, and **8**, recorded in THF. The spectrum of **8** was recorded at 60 °C; all other spectra were recorded at 25 °C. Dotted lines highlight the red-shifting effect of non-peripheral dodecyl substituents. The *Y*-axis represents molar extinction coefficients in units of M^−1^ cm^−1^. (**Right**) Beer–lambert law, A = εCl, is valid for the Q-band for all compounds, at least up to the indicated concentrations at the recording temperature.

Figure 2 contains the UV–vis spectra for **3**, **4**, **6**, **7**, and **8** recorded in THF. Supporting Information Figures S15–S19 shows UV–vis spectra for these compounds at different concentrations. Poor solubility of **8** required that its spectrum be recorded at 60 °C, while other spectra could be recorded at 25 °C. Results extracted from these UV–vis spectra are summarised in Table 1.

The electronic spectra of **3** and **8** have two absorption maxima in the Q-band region, Figure 2. This result is attributed to the D_2h_ compound symmetry. For **3**, the Q-band components are separated by Δλ = λ_max Qy_ − λ_max Qx_ = 31 nm, while, for **8**, the separation is Δλ = λ_max Qy_ − λ_max Qx_ = 36 nm. Phthalocyanines **4**, **6**, and **7** have D_4h_ symmetry. As a result, only one Q-band absorption maximum is observed. D_4h_ symmetry differs from D_2h_ symmetry in that the lowest energy singlet state is degenerate, causing the Q_x_ and Q_y_ components to merge into a single absorption maxima [4].

For metal-free phthalocyanines, changing dodecyl substituents from peripheral to non-peripheral positions redshifts the Q_x_ λ_max_ by Δλ_max Qx_ = λ_max Qx, **3**_ − λ_max Qx, **8**_ = 694 − 668 = 26 nm and Q_y_ λ_max_ by Δλ_max Qy_ = λ_max Qy, **3**_ − λ_max Qy, **8**_ = 725 − 704 = 21 nm (Table 1). The non-peripheral substitution pattern redshifts the zinc-containing phthalocyanine Q-band maximum of **4** by Δλ_max_ = λ_max **4**_ − λ_max **6**_ = 698 − 679 = 19 nm, compared to the peripheral substitution pattern of **6**. This renders **4** a stronger candidate for photodynamic therapy than **6**, as light achieves deeper tissue penetration at this wavelength [27].

Adherence of the Q-bands to the Beer–Lambert law (Figure 2) implies that all phthalocyanines remain unaggregated up to at least 6 µM in THF at the spectrum recording temperature. Figures S16–S20 in the Supporting Information show that the Soret band also follows the Beer–Lambert law.

Q-band molar extinction coefficients of octa-substituted alkoxy and thioalkyl metal-free phthalocyanines have previously been reported, within the range of 41,100–135,600 M^−1^ cm^−1^, while their zinc derivatives have Q-band ε-values between 139,100 and 248,800 M^−1^ cm^−1^ [15]. These literature value ranges are comparable with that obtained for the octa-dodecyl metal-free (121,000 ≤ ε (Q_y_) ≤ 177,000 M^−1^ cm^−1^) and zinc phthalocyanines (193,000 ≤ ε ≤ 204,000 M^−1^ cm^−1^) presented in this work.

### 2.4. X-ray Photoelectron Spectroscopy

**Figure 3:**
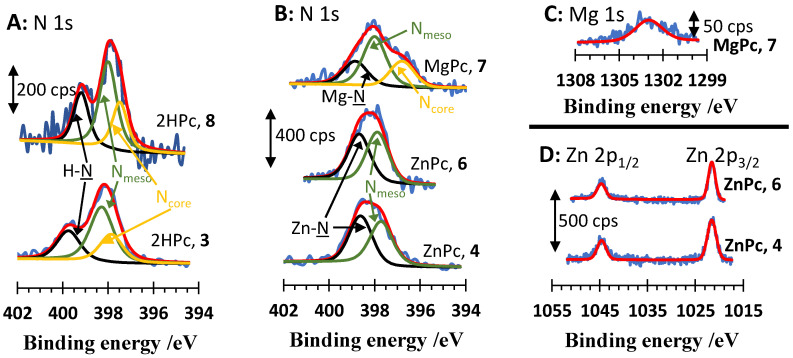
(**Left**, **A**) Nitrogen 1s XPS envelopes of metal-free phthalocyanines **3** and **8**. (**Middle**, **B**) Nitrogen 1s XPS envelopes of metalated phthalocyanines **4**, **6**, and **7**. (**Right**, **C**) Mg 1s envelope for **7**. (**Right**, **D**) Zinc 2p envelopes for phthalocyanines **4** and **6**. In all spectra, experimental photoelectron lines are blue, and simulated Gaussian photoelectron lines are red. For **A** and **B**, black lines represent either the simulated N–H, N–Mg, or N–Zn photoelectron lines, green lines represent simulated N_meso_ photoelectron lines, while, for **A** and **B** (top), orange lines represent N_core_ photoelectron lines. In **B**, for **7**, the four Mg–N bonds are not considered equivalent, see Scheme 1. Rather, due to the small size of the Mg^2+^ cation, they are considered as two inequivalent sets of Mg–N bonding interactions. One set is considered strong bonding interactions (the black Mg–N photoelectron line), while the other set is considered to be weak bonding interactions (the orange photoelectron line labelled N_core_).

Figure 3 displays the X-ray photoelectron spectra (XPS) of the nitrogen 1s, magnesium 1s, and zinc 2p envelopes for all phthalocyanines. Data extracted from Figure 3 is summarised in Table 2.

Each phthalocyanine has four meso nitrogen atoms (N_meso_) and four core nitrogens. For metal-free phthalocyanines (**3** and **8**), two of the core nitrogens are bound to hydrogen atoms (N–H), while the remainder exist as core nitrogen components, N_core_, of the phthalocyanine macrocycle.

Metal derivatives (**4** and **6**) differ, in this regard, in that all four core nitrogens are symmetrically coordinated to the metal centre (N-M). Experimental N 1s XPS spectra could not resolve these different nitrogen atom types unambiguously (Figure 3). However, the binding energies (BE) for these different nitrogens could be estimated by simulating the experimental spectra through Gaussian curve fitting (Table 2). Three Gaussian curves, with a fixed area ratio of 1:2:1, were used for metal-free phthalocyanines, while two Gaussian curves, with a fixed area ratio of 1:1, were used for the metal derivatives **4** and **6**. The N 1s envelope of the magnesium derivative **7** differed from those of the zinc derivatives **4** and **6**, in that, for **7**, similar to the metal-free derivatives, two different photoelectron lines needed to be simulated to replicate the Mg–N_core_ experimental photoelectron lines. To explain this, it is noted that Zn^2+^ ions have an ionic radius of 0.74 Å, while Mg^2+^ ions have an ionic radius of 0.65 Å [28]. This, together with the XPS result, showing two sets of Mg–N photoelectron lines of equal intensity, is consistent with an asymmetric bonding pattern, as shown for **7** in Scheme 1.

Moving from a peripheral to non-peripheral substitution pattern in metal-free phthalocyanines **3** and **8** increases the binding energy of N_meso_, N–H, and N_core_ by 0.3 eV (an insignificant change, a BE change of ≥0.4 eV is considered significant), 0.6 eV, and 0.4 eV, respectively (the latter is on the extreme edge of being significant). For zinc phthalocyanines **4** and **6**, the change in substitution pattern has no significant influence on the binding energy for N_meso_ and N_core_ atoms (0.2 eV and 0.1 eV difference, respectively, Table 2). Zinc insertion in the non-peripheral series (**3** and **4**) has a marginally significant influence on the binding energy of N_meso_ (397.7 − 398.3 = −0.6 eV), whereas both zinc and magnesium insertion into the peripheral series (i.e., comparing **8** with **6** and **7**) has a negligible N_meso_ binding energy difference.

No significant XPS photoelectron line shifts are noted for the Zn 2p envelope, when comparing the non-peripherally (**4**) and peripherally (**6**) substituted zinc phthalocyanines (Figure 3, Table 2).

### 2.5. Electrochemistry

**Figure 4:**
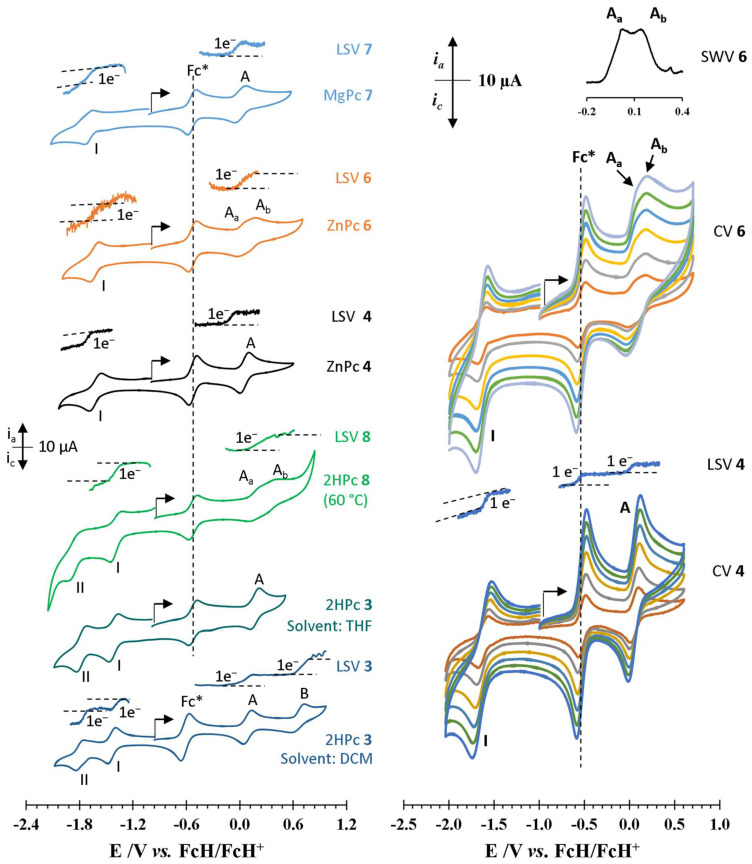
Left: Cyclic voltammograms (CVs) of the indicated phthalocyanines at a scan rate of 100 mV/s in THF or for **3** only, in DCM at 25 °C unless otherwise indicated. Selected linear sweep voltammograms (LSVs), recorded at a scan rate of 1 mV/s, are also shown. Right: CVs of **4** and **6** at scan rates of 50 (smallest currents), 100, 200, 300, 400, and 500 mV/s (biggest currents) in THF at 25 °C. Inserts: Square wave voltammogram (SWV) of the first ring-based oxidation of **6**, recorded at 20 Hz, in THF at 25 °C, highlighting better resolution between waves A_a_ and A_b_ and linear sweep voltammograms (LSVs) of **4**, indicating the relative number of electrons involved in each redox process.

Selected cyclic voltammograms (CVs) of phthalocyanines **3**, **4**, **6**, **7**, and **8** are displayed in Figure 4. Cyclic voltammetric data, applicable to these CVs, are summarised in Table 3. Due to the poor solubility of especially **8**, CVs of all phthalocyanines were recorded in tetrahydrofuran (THF), either at 60 (only **8**) or 25 °C (all other phthalocyanines). The cyclic voltammetry of **3** was also studied in dichloromethane (DCM) at 25 °C. Supporting Information Figures S20–S24 and Tables S1–S6 contains CVs and data at different scan rates.

Electrochemical reversibility for one-electron transfer processes is characterised by ΔE_p_ = E_pa_ − E_pc_ = 59 mV [29]. All redox processes labelled A (anodic waves), I, and II (cathodic waves) for **3**, **4**, and **6**–**8** exhibit quasi electrochemical reversibility at slow scan rates, with 78 < ΔE_pa_ < 135 mV (Table 3), but approach chemical reversibility (i.e., current ratios approached unity) with 0.83 < *i_pa_*/*i_pc_* < 1.00. In the THF potential window, only one of the two possible oxidation couples, wave A, was observed. As is well-known and -documented in literature [3,7,15], these phthalocyanine ring-based redox processes are all one-electron transfer processes. In this study, we report the linear sweep voltammetry (LSV) of compounds **3**, **4**, **6**, **7**, and **8** in Figure 4. The LSV show that the number of electrons transferred is the same for the observed oxidation and reduction waves; since it is known that the phthalocyanines ring-based processes are one-electron processes, it can also be extrapolated to all main waves of the present compounds studied. In the case of compound **8**, the combined wave A_a_ and A_b_ gives an LSV wave of equal size to that of the other one-electron reduction wave. The LSV of the second reduction wave, wave II, is not always shown, since decomposition in many cases occurred on LSV time-scale, and wave II could not be observed on the LSV scans. Additionally, the peak current (*i_p_*) values of ring-based processes are all of approximately equal size, as can be seen from Figure 4 and Table 3. Peak currents are the analytical quantity measured for concentration in the Randles Cevcik equation. Together with the LSV results, peak current values are, therefore, also mutually consistent with all ring-based oxidation and reduction waves being one-electron transfer processes.

The peripherally substituted phthalocyanines **6** and **8** display CV features consistent with dimerization, as wave A is split into two components, A_a_ and A_b_, of approximately equal intensity, with Δ*E*°′_Aa,Ab_ = *E*°′_Aa_ − *E*°′_Ab_ = ca. 195 mV for **8** or 118 mV for **6** (Table 3 and Figure 4) [30,31]. Wave A, of the peripherally substituted Mg complex **7**, displays weak evidence of dimerization (see enlarged CV in Supporting Information, Figure S24), but the resolution between A_a_ and A_b_ is too poor to assign component potentials and currents. As a result, Table 3 only reports observed peak currents for this phthalocyanine.

**Scheme 2:**
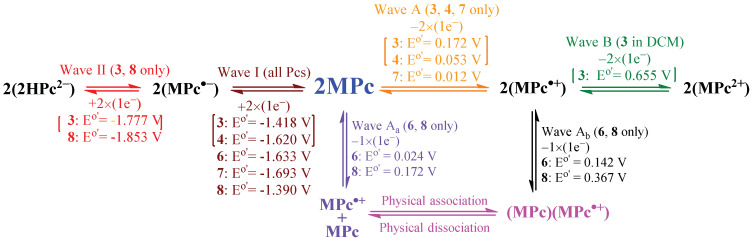
Proposed electrochemical mechanism consistent with the observed redox processes for **3**, **4**, **6**, **7**, and **8**. Non-peripherally substituted phthalocyanines are associated with brackets ([]), whereas peripheral substituted phthalocyanines are not. Wave B was only observed for **3** in DCM (dichloromethane). MPc represents the ground state. E°′ values cited are those determined in THF, unless otherwise stated.

Peak splitting into two wave A components for the non-peripheral complexes **3** and **4** is not observed. To understand why peripherally substituted phthalocyanines exhibit dimerization features in wave A, but non-peripherally substituted derivatives do not, consideration of the structural differences between peripherally and non-peripherally substituted complexes, as described at the end of Section 2.1, are instructive. From the crystal structure of non-peripheral octakisalkylphthalocyanine complexes, inter-macrocyclic distances are ca. 8.5 Å [25], while, for peripheral substituted complexes, it is about 3.6 Å [5,26]. The stronger core:core interactions that exist in peripherally substituted phthalocyanines, which result from a closer inter-macrocyclic distance, imply that oxidized phthalocyaninato species having a positive charge (i.e., they are electrophilic) may easily form dimeric intermediates with their neutral (i.e., unoxidized and nucleophilic) counterparts, see Scheme 2.

Phthalocyanine **3** was soluble enough in dichloromethane (DCM) to allow cyclic voltammetry therein. The solvent window of DCM has a larger positive potential limit than that of THF. This allowed for the observation of the second one electron, ring-based oxidation of **3**, wave B (Figure 4). This second anodic process is both electrochemically and chemically irreversible, with Δ*E*_p_ = 143 mV and *i_pc_/i_pa_* = 0.56, Table 3.

Scheme 2 illustrates an electrochemical mechanism related to the results that were obtained. From Scheme 2, several observations can be made. For metal-free phthalocyanines **3** and **8**, changing from non-peripheral substituents to a peripheral substitution pattern decreased the E°′ of cathodic wave II by Δ*E*°′_wave II_ = *E*°′**_8_**_, wave II_ − *E*°′**_3_**_, wave II_ = −1.853 − (−1.800) = −0.053 V. Contrary to wave II, the E°′ for cathodic wave I of the peripherally substituted compound **8** increased slightly with 28 mV, when compared to the non-peripheral derivative **3**. The first oxidation wave of the peripherally substituted metal-free phthalocyanine (A_a_ of **8**) had the same E°′ as that of the non-peripheral complex (A of **3**), with *E*°′ = 0.172 V vs. FcH/FcH^+^.

Zinc metalation decreased the formal reduction potential of the reduction wave I of the non-peripheral (**3**, **4**) and peripheral series (**6**, **7**, **8**) by 202 mV and 243 mV, respectively. For the first oxidation redox process, zinc insertion decreased E°′_wave A_ of the non-peripherally substituted complex **4**, with 118 mV, relative to that of **3**, as well as for the peripherally substituted complex, zinc insertion lowered *E*°′ of wave A_a_, with 148 mV on moving from **8** to **6**. *E*°′ of the peripherally substituted zinc complex **6** was consistently slightly smaller than that of the non-peripherally substituted derivative **4**, with 13 mV for wave I and 30 mV for wave A/A_a_. Magnesium insertion into the peripheral series lowered the formal reduction potentials of **7** slightly more than zinc insertion, Table 3 and Scheme 2.

Phthalocyanine **7** had the highest HOMO-LUMO (HL) gap of all the phthalocyanines listed in Table 3, with Δ*E*°′_HL_ = 1.705 mV. For non-peripherally substituted phthalocyanines, the Δ*E*°′_HL_ increased by 1.673 – 1.590 = 0.083 V (Table 3) during zinc insertion on going from **3** to **4**, while, for the peripheral series, the Δ*E*°′_HL_ on going from **8** to **6** increased by 95 mV (wave A_a_ is used; 1.657–1.562 V). Non-peripherally substituted phthalocyanines have larger Δ*E*°′_HL_ in both the metal-free (1.590 vs. 1.562 V) and zinc complex (1.674 vs. 1.657 V).

## 3. Materials and Methods

### 3.1. General Procedures

All solid reagents were purchased from Sigma Aldrich, Johannesburg, South Africa and used without further purification. Liquid reagents (Sigma-Aldrich and Merck, Johannesburg, South Africa) were used, without any further purification unless stated otherwise. For electrochemical experiments, spectrochemical grade THF, or dichloromethane (Sigma-Aldrich) was used and stored under an argon atmosphere. Distilled water was used throughout. 3,6-Bis(dodecyl)phthalonitrile, **2** [3], and 4,5-bis(dodecyl)phthalonitrile, **5** [22,23], were synthesised using modified literature procedures (See Supporting Information for details). [N(^n^Bu_4_)][B(C_6_F_6_)_4_] was synthesized as described before [21]. Column chromatography was performed on Kieselgel 60 (Merck, grain size 0.040–0.063 nm) as the stationary phase.

### 3.2. Spectroscopic Characterization Techniques

#### 3.2.1. Nuclear Magnetic Resonance

Solution phase ^1^H NMR spectra were recorded on either a Bruker 400 MHz AVANCE III NMR spectrometer (Bruker, Johannesburg, South Africa), operating at 400.13 MHz and 25 °C or 50 °C, or on a Bruker 600 MHz AVANCE II NMR spectrometer (Bruker, Johannesburg, South Africa), operating at 600.28 MHz and 25 °C.

#### 3.2.2. UV–Vis Spectroscopy

UV-visible spectroscopy was performed in neat THF on a Varian Cary 60 (Varian, Johannesburg, South Africa) dual beam UV–Vis–NIR spectrometer, using a quartz cell, with a 1 cm path length at either 25 °C or 60 °C.

#### 3.2.3. Attenuated Total Reflectance Fourier Transform Infrared Spectroscopy

ATR–FTIR spectra were collected on a Bruker Tensor 27 FT-IR spectrometer (Bruker, Johannesburg, South Africa), equipped with a PIKE MIRacle ATR attachment (Bruker, Johannesburg, South Africa).

#### 3.2.4. X-ray Photoelectron Spectroscopy

XPS spectra were recorded on a PHI 5000 Versaprobe (Ulvac-Phi, Chigasakie, Japan) system utilizing a monochromatic Al K_α_ X-ray source (h*υ* = 1253.6 eV). Details, relating to the XPS analysis, are similar to other XPS analysis reported from our laboratory [32].

### 3.3. Synthesis

Scheme 1 illustrates the reaction paths to obtain compounds **3**, **4**, **6**–**8**.

#### 3.3.1. 1,4,8,11,15,18,22,25-Octakis(dodecyl)phthalocyanine, **3**

To 3,6-bis(dodecyl)phthalonitrile, **2**, (740 mg, 1.6 mmol) dissolved in n-pentanol (8 mL), at 80 °C under argon, was added clean lithium metal fragments (0.3 g, 43 mmol). The mixture was then heated at 110 °C for 18 h, allowed to cool to room temperature, and treated with acetone (70 mL). After 30 min, the solution was filtered, and the remaining solids were extracted with acetone (2 × 50 mL). The combined acetone fractions were concentrated to ca 30 mL, after which acetic acid (40 mL) was added, and the suspension was allowed to stir for 30 min. Filtration and drying gave the crude metal-free phthalocyanine in a yield of 204 mg. The crude phthalocyanine product was then subjected to column chromatography using an eluent ratio of DCM/n-hexane 1:4 to elute **3**. Precipitation of the recovered product from THF by the dropwise addition of methanol gave phthalocyanine **3** as a green solid (149 mg, 0.08 mmol, 20% yield), m. p. 115 °C. ATR FTIR/cm^−1^: *υ*(C-H) 2849, *υ*(C-H) 2917, *υ*(N–H) 3294. ^1^H NMR (CDCl_3_, 400 MHz)/ppm: 7.87 (s, 8H), 4.45 (t, 16H, *J* = 7.4 Hz), 2.08 (m, 16H, *J* = 7.6 Hz), 1.33 (m, 16H, *J* = 7.0 Hz), 1.16 (s, 128H), 0.82 (t, 24H, *J* = 7.0 Hz). Anal. Calcd for C_128_H_210_N_8_: C, 82.6; H, 11.4; N, 6.0. Found: C, 82.2; H, 11.2; N, 5.6 [3].

#### 3.3.2. 1,4,8,11,15,18,22,25-Octakis(dodecyl)phthalocyaninatozinc(II), **4**

The metal-free phthalocyanine, **3**, (50 mg, 0.027 mmol), anhydrous zinc acetate (24 mg, 0.11 mmol), and n-pentanol (2.7 mL) was stirred at 130 °C for 4 h under argon. After cooling to room temperature, the mixture was treated with methanol (10 mL), and the resulting precipitate filtered. The recovered precipitate was washed with methanol (3 × 20 mL), after which, the crude zinc phthalocyanine was purified by fractional precipitation from THF/methanol to yield **4** as a blue solid (47 mg, 0.024 mmol, 89% yield), m. p. 210 °C. ATR FTIR/cm^−1^: *υ*(C-H) 2849, *υ*(C-H) 2917. ^1^H NMR (THF-*d*_8_, 600 MHz)/ppm: 7.88 (s, 8H), 4.62 (t, 16H, *J* = 7.36 Hz), 2.21 (m, 16H, *J* = 7.5 Hz), 1.63 (m, 16H, *J* = 7.6 Hz), 1.36 (m, 16H, *J* = 7.4 Hz), 1.18 (s, 112H), 0.82 (t, 24H, *J* = 7.08 Hz). Anal. Calcd for C_128_H_208_N_8_Zn: C, 79.9; H, 10.9; N, 5.8. Found: C, 79.8; H, 10.9; N, 5.6 [3].

#### 3.3.3. 2,3,9,10,16,17,23,24-Octakis(dodecyl)phthalocyaninatozinc(II), **6**

Anhydrous zinc acetate (100 mg, 0.54 mmol), **5** (225 mg, 0.48 mmol), and freshly distilled dimethylaminoethanol (5 mL) was heated at 130 °C for 16 h in the dark under argon while stirring. The mixture was allowed to cool to room temperature, solvent removed under reduced pressure, and remaining residue washed with methanol/water (5/1). After drying, the crude zinc phthalocyanine was chromatographed over silica gel with THF/toluene 1:10 as eluent. The first band afforded after solvent removal and precipitation from THF by the dropwise addition of methanol **6** as a blue solid (25 mg, 0.013 mmol, 11% yield), m. p. 315 °C. ATR FTIR/cm^−1^: *υ*(C-H) 2849, *υ*(C-H) 2918. ^1^H NMR (THF-*d*_8_, 400 MHz)/ppm: 9.23 (s, 8H), 3.25 (q, 16H, *J* = 8.0 Hz), 2.04 (m, 16H, *J* = 7.7 Hz), 1.58 (t, 16H, *J* = 10.8 Hz), 1.30 (s, 128H), 0.88 (t, 24H, *J* = 6.8 Hz). Anal. Calcd for C_128_H_208_N_8_Zn: C, 79.9; H, 10.9; N, 5.8. Found: C, 79.6; H, 10.7; N 5.5.

#### 3.3.4. 2,3,9,10,16,17,23,24-Octakis(dodecyl)phthalocyaninatomagnesium(II), **7**

Mg(OAc)_2_·4H_2_O (50 mg, 0.23 mmol) and **5** (200 mg, 0.43 mmol) was dissolved in 5 mL of n-pentanol under argon. DBU (100 µL) was added, after which, the mixture was refluxed at 130 °C for 18 h in the dark. Methanol (10 mL) was added to the cooled solution, and the resulting blue precipitate was first filtered and then washed with methanol (10 mL). Fractional precipitation from THF/methanol was followed by chromatography over silica, using THF as eluent. A final precipitation from the dropwise addition of methanol to a THF solution of the crude product yielded **7** as a blue solid (78.4 mg, 0.042 mmol, 39% yield), m. p. 261 °C. ATR FTIR/cm^−1^: *υ*(C-H) 2849, *υ*(C-H) 2918. ^1^H NMR (THF-*d*_8_, 400 MHz)/ppm: 9.25 (s, 8H), 3.26 (q, 16H, *J* = 5.3 Hz), 2.05 (m, 16H, *J* = 6.2 Hz), 1.58 (q, 16H, *J* = 7.33 Hz), 1.30 (s, 128H), 0.88 (t, 24H, *J* = 6.9 Hz). Anal. Calcd for C_128_H_208_N_8_Mg: C, 81.6; H, 11.1; N, 6.0; Found: C, 81.4; H, 10.9; N, 5.7.

#### 3.3.5. 2,3,9,10,16,17,23,24-Octakis(dodecyl)phthalocyanine, **8**

*By cyclisation of***5**: Phthalonitrile **5** (200 mg, 0.43 mmol) was dissolved in warm n-pentanol (2.5 mL) under argon, followed by the addition of clean lithium metal fragments (0.15 g, 22 mmol). After heating at 110 °C for 16 h, the mixture was cooled, and the solid residue extracted with warm acetone (60 mL). The extract was filtered, concentrated to *ca* 25 mL, and treated with 40 mL of acetic acid. After stirring for 30 min, methanol (40 mL) was added. The fine precipitate was collected by centrifuging at 9500× *g*, followed by washing with methanol (2 × 40 mL), drying, and precipitation from warm THF (ca 60 °C) by the dropwise addition of methanol to yield **8** as a blue solid (20.2 mg, 0.011 mmol, 10% yield), m. p. 247 °C. ATR FTIR/cm^−1^: *υ*(C-H) 2848, *υ*(C-H) 2917, *υ*(N–H) 3358. ^1^H NMR (THF-*d*_8_, 600 MHz)/ppm: 9.04 (s, 8H), 3.24 (t, 16H, *J* = 8.27 Hz), 2.06 (m, 16H, *J* = 4.4 Hz), 1.60 (m, 16H, *J* = 5.9 Hz), 1.26 (s, 128H), 0.87 (t, 24H, *J* = 7.0 Hz). Anal. Calcd for C_128_H_210_N_8_: C, 82.6; H, 11.4; N, 6.0. Found: C, 82.4; H, 11.1; N, 5.7.

*Magnesium demetalation route*: The magnesium phthalocyanine, **7**, (20 mg, 0.011 mmol), glacial acetic acid (3 mL), and n-pentanol (3 mL) were heated at 130 °C for 0.5 h under argon. Methanol (10 mL) was added to the cooled solution, after which, the precipitate was collected by means of filtration. The residue was washed with methanol (2 × 30 mL), dissolved in warm (ca 60 °C) THF, and reprecipitated by the dropwise addition of methanol. The precipitated solid was filtered and allowed to dry to yield **8**, as a dark blue solid (16.2 mg, 0.009 mmol, 82% yield). Characterisation data as above.

*Zinc demetalation route:* The zinc phthalocyanine, **6**, (30 mg, 0.015 mmol), pyridine hydrochloride (1 g), and freshly distilled pyridine (2 mL) were heated at 120 °C for 20 h under argon. Heating was halted and distilled water (10 mL) was added to the warm reaction mixture. Filtration followed by washing with water (3 × 30 mL) and methanol (3 × 30 mL) isolated the crude metal free phthalocyanine, which was further purified by washing with cold tetrahydrofuran and then reprecipitation from warm THF (ca 60 °C), with methanol to yield **8**, as a light blue solid (23 mg, 0.012 mmol, 82% yield). Characterisation data as above.

### 3.4. Electrochemistry

Cyclic voltammetry, square wave voltammetry and linear sweep voltammetry were recorded using a PARSTAT^®^ 2273 potentiostat (Ametek Scientific Instruments, Oak Ridge, TN, USA), with the aid of Powersuite data collection software (Ametek Scientific Instruments, Oak Ridge, TN, USA). Electrochemical measurements were performed in either spectrochemical grade dichloromethane or tetrahydrofuran using 0.1 M [N(^n^Bu_4_)][B(C_6_F_6_)_4_], as supporting electrolyte and ca 0.5 mM decamethylferrocene (STREM, Newburyport, MA, USA), as internal reference, under an argon atmosphere. A platinum *pseudo* reference electrode, a platinum auxiliary electrode and a glassy carbon working electrode (active working area of 0.78 cm^2^) (Analytical Science Technology, Cape Town, South Africa) were employed. Potentials were referenced experimentally against decamethylferrocene, as internal standard, and were then manipulated on a spreadsheet, where *E*°′_ferrocene_ was set to 0 mV to report potentials as vs. FcH/FcH^+^. Decamethylferrocene has *E*°′ = −622 mV vs. FcH/FcH^+^ with ΔE = 74 mV in dichloromethane and *E*°′ = −527 mV vs. FcH/FcH^+^ with ΔE = 76 mV in tetrahydrofuran under the experimental conditions used.

## 4. Conclusions

Non-peripherally and peripherally octakis(dodecyl)-substituted phthalocyanines **3**, **4**, **6**, **7**, and **8** may be synthesized utilizing the same experimental techniques, but poorer solubility of the peripherally substituted derivatives complicate workup procedures, to the extent that columns cannot be run with the metal-free derivative **8**. This is also the reason demetalation of magnesium phthalocyanine **7** is a more efficient synthetic route to obtain **8** over cyclotetramerization of 4,5-bis(dodecyl)phthalonitrile.

Spectroscopically, the most striking ^1^H NMR differences was found between non-peripheral aromatic hydrogens of **6** and **7**, having resonances positions of 9.23 and 9.25 ppm, respectively, and the ^1^H NMR resonance of peripheral hydrogens of **4** is at 7.88 ppm, both in THF-*d*_8_. This is a drift of ca. 1.36 ppm. The difference in dodecyl substitution pattern also had an effect on the ^1^H NMR resonance position of the “benzylic” methylene protons (i.e., protons of the CH_2_ groups bound directly to the phthalocyanine core). Changing from a non-peripheral to a peripheral substitution pattern shifted the benzylic CH_2_ resonance of metal-free and zinc phthalocyanines **8** and **6** upfield by more than 1.21 ppm, compared to the same resonance of **3** and **4**. Both resonance drifts are brought about by the steric hindrance imposed upon non-peripheral aromatic hydrogens and “benzylic” hydrogens of phthalocyanines **6** and **7** by the neighbouring peripheral dodecyl groups and adjacent phthalocyanine macrocycle. The peripheral hydrogens of **4** and the “benzylic” hydrogens of **3** and **4** are all more exposed (“naked”) and less deshielded, due to the differences in non-peripheral versus peripheral substitution patterns.

FTIR–ATR spectroscopy showed that this technique may be used to follow cyclotetramerization reactions of phthalonitriles by monitoring the disappearance of CN stretching vibrations at ca. 2229 cm^−1^. Metal-coordination reactions may be monitored by the disappearance of metal-free phthalocyanine N–H stretching vibrations at ca. 3300 cm^−1^.

In the electronic spectra of zinc phthalocyanines **4** and **6**, the Q-band maximum absorption wavelength, λ_max_, of non-peripherally substituted derivative **4** was red-shifted to 698 nm, compared to Q-band λ_max_ = 679 of the peripherally substituted derivative **6**. Because light of this wavelength penetrates deeper through body tissue, it follows that non-peripherally substituted zinc phthalocyanines may potentially be more suitable for photodynamic cancer therapy than their peripherally substituted counterparts.

X-ray photoelectron spectroscopy differentiated between N_H_, N_meso_ and N_core_ nitrogen atoms for metal-free phthalocyanines. Zinc phthalocyanines **4** and **6** have four equivalent N_meso_ and four equivalent N_Zn_ core nitrogen atoms. The unexpected result was for the Mg phthalocyanine **7** which, like metal-free phthalocyanines, also exhibited two sets of core N atoms. The smallness of the Mg^2+^ cation (ionic radius = 0.65 Å, compared to 0.74 Å for Zn^2+^) led to the conclusion that Mg is not centred in the phthalocyanine cavity and one photoelectron line set is associated with two core N atoms, strongly coordinated to Mg; the other set is associated with the two remaining N_core_ atoms, which are only involved with weak association with Mg^2+^.

Peripherally substituted phthalocyanines **6**, **7**, and **8** have cyclic voltammetry features, consistent with dimerization upon oxidation. No dimerization was observed during oxidation of non-peripherally substituted phthalocyanines **3** and **4**. Dimerization is a consequence of the closer distance between peripherally substituted macrocycles, compared to the distance between non-peripherally substituted phthalocyaninato macrocyclic rings, 3.6 Å versus 8.5 Å. The closer contact distance between peripherally substituted macrocycles is conducive for nucleophilic–electrophilic macrocycle pair formation, leading to intermediate structures, such as [Pc][Pc^+^]. Metalation of metal-free peripherally, as well as non-peripherally substituted phthalocyanines, shifted the formal reduction potentials of all redox processes to lower potentials because metal insertion leaves the macrocycles more electron rich. This conclusion is consistent with the electronegativity of two hydrogen atoms, being 2 × 2.2 = 4.4, while (from any periodic table) the electronegativity of one Zn or Mg atom is only 1.7 and 1.2, respectively. Hence, less work is required to remove electrons during oxidation half cycles from the metallated phthalocyaninato macrocyclic core.

## Figures and Tables

**Table 1:** Q-band λ_max_ and log*ε* data for **3**, **4**, **6**, **7**, and **8**.

Compound	*λ*_max_/nm (log*ε*) ^a^		
	Q	Q_x_	Q_y_
2HPc, **3**	-	694 (5.01)	725 (5.08)
ZnPc, **4**	698 (5.31)	-	-
ZnPc, **6**	679 (5.29)	-	-
MgPc, **7**	681 (5.50)	-	-
2HPc, **8**	-	668 (5.20)	704 (5.25)

^a^ ***ε*** = extinction coefficient in units of M^−1^ cm^−1^.

**Table 2:** Binding energies (BE) and atomic % for the nitrogen 1s, zinc 2p, and magnesium 1s envelopes.

Compound	N_meso_ 1s BE/eV (Atomic %)	N-M; N_core_ BE/eV (Atomic %)	Metal 1s or 2p BE/eV (Atomic %)	Molecular Stoichiometry ^e^ Experimental (Theoretical)
**3** (M = 2H)	398.3 (1.77)	399.8 (0.88); 397.9 (0.88)	-	N_meso_:N_H_:N_core_ = 4:2:2 ^f^ (4:2:2) ^g^
**4** (M = Zn)	397.7 (2.25)	398.6 (2.25); -	1044.6 ^a^; 1021.6 ^b^ (0.55) ^c^	N_meso_:N_Zn_:Zn = 4:4:0.98 ^f^ (4:4:1) ^g^
**6** (M = Zn)	397.9 (2.39)	398.7 (2.39); -	1044.7 ^a^; 1021.6 ^b^ (0.59) ^c^	N_meso_:N_Zn_:Zn = 4:4:0.99 ^f^ (4:4:1) ^g^
**7** (M = Mg)	398.0 (2.18)	398.9 (1.09); 396.7 (1.09)	1303.0 ^d^ (0.51)	N_meso_:N_Zn_:N_core_:Mg = 4:2:2:0.94 ^f^ (4:2:2:1) ^g^
**8** (M = 2H)	398.0 (1.23)	399.2 (0.61); 397.5 (0.61)	-	N_meso_:N_H_:N_core_ = 4:2:2 ^f^ (4:2:2) ^g^

^a^ Zn 2p_1/2_; ^b^ Zn 2p_3/2_; ^c^ of Zn 2p_3/2_ ^d^ Mg 1s; ^e^ of the indicated atoms only; ^f^ experimental atom ratios; ^g^ theoretical atom ratios.

**Table 3:** Peak anodic potentials, *E*_pa_, peak potential differences, Δ*E*_p_, formal reduction potentials, E°′, peak anodic or cathodic currents, *i_pa_* and *i_pc_*, respectively, and current ratio, *i_pc_/i_pa_* or *i_pa_/i_pc_* for the 100 mV/s scan rate redox couples, indicated in Figure 4.

Wave	*E*_pa_/V	Δ*E*_p_/mV ^a^	*E*°′/V	*i_p_*_a_/μA	*i*_pc_/*i*_pa_	Wave	*E*_pa_/V	Δ*E*_p_/mV ^a^	*E*°′/V	*i*_pa_/μA	*i*_pc_/*i*_pa_
Decamethylferrocene (THF)						Ferrocene (THF)					
-	−0.488	76	−0.527	6.00	0.96	-	0.039	78	0.000	10.80	0.98
Decamethylferrocene (DCM)						Ferrocene (DCM)					
-	−0.575	74	−0.614	5.00	0.98	-	0.035	70	0.000	6.50	0.96
3 (2H, non-peripheral, DCM) Δ*E*°′_HL_ = 1.531 V ^b^						8 (2H, peripheral, THF) Δ*E*°′_HL_ = 1.562 V ^b^					
**A**	0.136	97	0.088	4.75	1.00	**A_a_**	0.227 ^f^	111	0.172	1.56	0.83
**B**	0.726	143	0.655 ^c^	3.38	0.56	**A_b_**	0.416 ^f^	98	0.367	1.80	0.83
**I**	−1.396	93	−1.443	4.25 ^d^	0.97 ^e^	**I**	−1.326	127	−1.390	3.44 ^d^	0.82 ^e^
**II**	−1.754	92	−1.800	3.13 ^d^	0.96 ^e^	**II**	−1.785	135	−1.853	2.50 ^d^	1.00 ^e^
3 (2H, non-peripheral, THF) Δ*E*°′_HL_ = 1.590 V ^b^						6 (Zn, peripheral, THF) Δ*E*°′_HL_ = 1.657 V ^b^					
**A**	0.221	99	0.172	3.40	0.90	**A_a_**	0.067 ^f^	86	0.024	1.90	0.93
**I**	−1.364	107	−1.418	3.40 ^d^	0.88 ^e^	**A_b_**	0.181 ^f^	78	0.142	1.67	1.00
**II**	−1.716	121	−1.777	3.40 ^d^	0.88 ^e^	**I**	−1.578	113	−1.633	3.10 ^d^	0.85 ^e^
4 (Zn, non-peripheral, THF) Δ*E*°′_HL_ = 1.673 V ^b^						7 (Mg, peripheral, THF) Δ*E*°′_HL_ = 1.705 V ^b^					
**A**	0.105	105	0.053	3.60	0.97	**A**	0.080	135	0.012	2.40	0.96
**I**	−1.556	128	−1.620	2.60 ^d^	1.00 ^e^	**I**	−1.646	95	−1.693	1.90 ^d^	0.95 ^e^

^a^ Δ*E*_p_ = *E*_pa_ − *E*_pc_. ^b^ Δ*E*°′_HL_ = *E*°′_wave A (or Aa)_ − *E*°′_wave I_. Δ*E*°′_HL_ = electrochemical HOMO-LUMO gap. ^c^ Strictly speaking, the Nernst equation, and equations derived therefrom, only hold true for electrochemically reversible processes, i.e., those that exhibit ΔE_p_ approaching 59 mV. ^d^ Actually *i_pc_*. ^e^ Because current ratios are defined as *i_reverse scan_/i_forward scan_*, this is actually *i_pa_/i_pc_*. ^f^ Due to poor peak resolution, values are only approximations.

## Data Availability

Supporting Information is available online.

## Supplementary Materials

The following supporting information can be downloaded online. Four-step experimental procedure to obtain precursor phthalonitrile **2** [3]; five-step experimental procedure to obtain precursor phthalonitrile **5** [22,24]; Figures S1–S13: NMR Spectra of all compounds and precursors; Figure S14: infra-red spectra of all compounds; Figures S15–S19: UV–vis spectra and Beer–Lambert relationships for **3**, **4**, **6**, **7**, and **8.** Figure S20: CVs of ferrocene and decamethylferrocene in THF and DCM, at scan rates 50–500 mV.s^−1^; Figure S21: CVs of **3** in DCM, at scan rates 50–500 mV.s^−1^; Figures S22–S24: CVs of **3**, **7**, and **8** in THF, at scan rates 50–500 mV.s^−1^; Tables S1–S6: CV data that can be extracted from these CVs. Scheme S1: Synthesis of 3,6-bis(dodecyl)phthalonitrile, **2**, from thiophene, **9**. Scheme S2: Synthesis of 4,5-bis(dodecyl)phthalonitrile, **5**, from phthalimide, **13**.

## Sample Availability

Samples of the compounds are not available from the authors.
