# Supplementary material for: Octakis(dodecyl)phthalocyanines: Influence of Peripheral versus Non-Peripheral Substitution on Synthetic Routes, Spectroscopy and Electrochemical Behaviour
Source: Molecules. 2022 Feb 24;27(5):1529. doi: 10.3390/molecules27051529 (PMC8911678; doi:10.3390/molecules27051529)
Supplement: Supplementary file 1 [file molecules-27-01529-s001.zip › molecules-1572415-supplementary.pdf]

Octakis(dodecyl)phthalocyanines: Influence of peripheral *versus* non-peripheral substitution on synthetic routes, spectroscopy and electrochemical behaviour

Glendin Swart, Eleanor Fourie* and Jannie C. Swarts

Department of Chemistry, University of the Free State, Bloemfontein 9300, South Africa; 2015271865@ufs4life.ac.za (G.S); swartsjc@ufs.ac.za (J.C.S)

* Correspondence: fouriee@ufs.ac.za; Tel.: Tel:+27-51-4012701; Fax: +27-51-4017295

Supplementary Information

A. EXPERIMENTAL SECTION, including Schemes S1 and S2………………… 2

B. ^{1}H NMR SPECTRA including Figures S1-S13…………………………………. 7

C. FOURIER TRANSFORM INFRA-RED ATTENUATED TOTAL REFLECTANCE SPECTRA including Figure S14………………… 14

D. UV-VIS SPECTROSCOPY including Figures S15-S20………………………... 15

E. ELECTROCHEMISTRY including Figures S20-S25 and Tables S1-S6………. 18

F. REFERENCES…………………………………………………………………. 23

A. EXPERIMENTAL SECTION

Materials: THF was pre-dried over sodium-benzophenone and distilled under argon prior to use; pyridine was pre-dried over KOH and distilled under argon prior to use; commercially available anhydrous dioxane was purchased from Sigma Aldrich and used without additional purification. All other reagents were purchased from Sigma Aldrich and used without additional purification.

Spectroscopy

Nuclear magnetic resonance spectroscopy. ^{1}H NMR spectra were recorded on either a Bruker 300 MHz FOURIER NMR spectrometer operating at 300.18 MHz and 25 °C or a Bruker 400 MHz AVANCE III NMR spectrometer operating at 400.13 MHz and 25 °C or a Bruker 600 MHz AVANCE II NMR spectrometer operating at 600.28 MHz and 25 °C. Chemical shifts are referenced according to residual solvent signals ($CDCl_3$ = 7.26 ppm; THF-d_8 = 1.73 ppm; DMSO-d_6 = 2.50 ppm).

Fourier transform attenuated total reflectance infra-red spectroscopy. FT-ATR-IR was performed on a Bruker Tensor 27 FTIR-ATR spectrometer equipped with a PIKE MIRacle ATR-attachment and operating with OPUS data collection software.

Liquid phase UV-vis spectroscopy. UV-vis spectra were recorded in neat THF on a Varian Cary 60 or 5000 dual beam UV-Vis-NIR spectrometer using a quartz cell having a pathlength of 1 cm. Spectra of **8** were recorded at 60 °C, while all other phthalocyanine spectra were recorded at 25 °C.

Electrochemical study. Cyclic voltammograms (CV's) were recorded using a PARSTAT ® 2273 potentiostat operating with Powersuite data collection software. Spectrochemical grade THF or dichloromethane was used as solvent while 0.1 M [n-Bu_4][$B(C_6F_6)_4$] was used as supporting electrolyte. Decamethylferrocene (*ca* 0.5 mM) was used as internal reference.

Two platinum wires were employed as auxiliary and *pseudo* reference electrodes, while the working electrode was glassy carbon (active surface area of 7.07 mm^2). All cyclic voltammograms are referenced against FcH/FcH$^+$ at 0 mV. Potentials were manipulated in an external spreadsheet so as to be referenced against FcH/FcH$^+$ at 0 mV. In THF, decamethylferrocene has $E^{o\prime}$ = -527 mV *vs*. FcH/FcH$^+$ with ΔE_p = 76 mV, while in DCM, $E^{o\prime}$ = -622 mV *vs*. FcH/FcH$^+$ with ΔE_p = 74 mV.

Synthesis

Synthesis [1] of **2**, **10**, **11**, and **12** is illustrated in Scheme S1.

Synthesis [2,3] of **5**, **14, 15, 16** and **17** is illustrated in Scheme S2.

Synthesis of 3,6-bis(dodecyl)phthalonitrile, ***2****.*

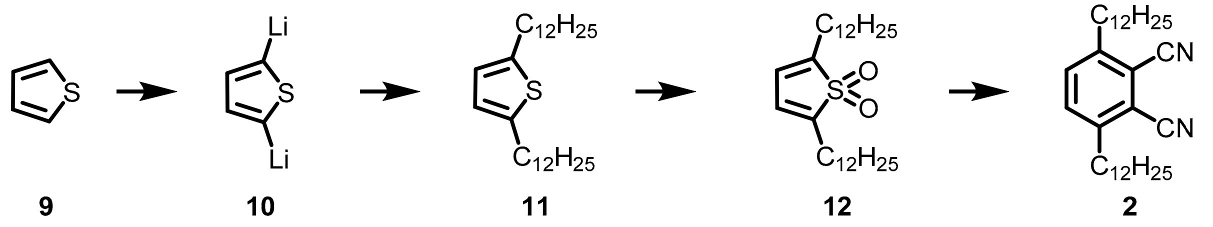

Scheme S1: Synthesis of 3,6-bis(dodecyl)phthalonitrile, **2**, from thiophene, **9**.

2,5-Bis(dodecyl)thiophene, ***11.*** n-Butyl lithium in hexane (250.0 ml, 1.6 M) was slowly added to a stirring solution of anhydrous THF (50 ml) and **9** (15 g, 0.18 mol) at -75 ºC under argon. After 24 hours, the reaction mixture was once again cooled to -75 ºC followed by the slow addition of 1-bromododecane (93 g, 0.38 mol, 90 ml). The reaction mixture was allowed to stir at room temperature for 24 hours, after which it was poured onto ice and extracted with diethyl ether. After drying ($MgSO_4$) and solvent removal, the remaining residue was recrystallized from absolute ethanol to yield **11** as an off-white solid (20 g, 4.8 mmol, 30 %

yield). ATR FTIR/cm^{-1}: υ(C-H) 2852, υ(C-H) 2917. ^{1}H NMR ($CDCl_3$, 300 MHz)/ppm: 6.55 (s, 2H), 2.73 (t, 4H, J = 7.6 Hz), 1.63 (m, 4H, J = 7.4 Hz), 1.26 (s, 36H), 0.88 (t, 6H, J = 6.7 Hz).

2,5-Bis(dodecyl)thiophene-1,1-dioxide, ***12.*** To a stirring heterogeneous mixture of water (500 ml), acetone (450 ml) and $NaHCO_3$ (500 g, 6 mol) was added **11** (18 g, 43 mmol) in 300 ml of DCM. The reaction mixture was cooled to 0 ºC, after which solid oxone ($2KHSO_5{\cdot}KHSO_4{\cdot}K_2SO_4$, 500 g, 0.8 mol) was added in small portions. After 2 days of stirring, the solution was first diluted with 2 l of water and then extracted with chloroform (1.5 l). Crystallization after drying ($MgSO_4$) and solvent removal from absolute ethanol afforded **12** as an off-white solid (14.5 g, 32.0 mmol, 67 % yield). ATR FTIR/cm^{-1}: υ(S=O) 1284, υ(C-H) 2852, υ(C-H) 2917. ^{1}H NMR ($CDCl_3$, 300 MHz)/ppm: 6.26 (s, 2H), 2.46 (t, 4H, J = 7.6 Hz), 1.63 (m, 4H, J = 7.6 Hz), 1.25 (s, 36H), 0.88 (t, 6H, J = 6.7 Hz).

3,6-Bis(dodecyl)phthalonitrile, ***2.*** Fumaronitrile (0.35 g, 4.4 mmol) and **12** (2 g, 4.4 mmol) were sealed with a minimum amount of chloroform (*ca* 0.3 ml) under argon. The stirring mixture was heated at 160 ºC for 24 hours, after which the residue was dissolved in chloroform and subjected to heating at 90 ºC and 50 mmHg pressure. Column chromatography over silica using pure toluene (R_f = 0.9), followed by recrystallization from absolute ethanol yielded **2** as an off-white solid (0.59 g, 1.3 mmol, 29 % yield). ATR FTIR/cm^{-1}: υ(C≡N) 2228, υ(C-H) 2848, υ(C-H) 2943. ^{1}H NMR ($CDCl_3$, 300 MHz)/ppm: 7.45 (s, 2H), 2.84 (t, 4H, J = 7.8 Hz), 1.64 (m, 4H, J = 7.3 Hz), 1.25 (s, 36H), 0.88 (t, 6H, J = 6.7 Hz).

Synthesis of 4,5-bis(dodecyl)phthalonitrile, ***5,***

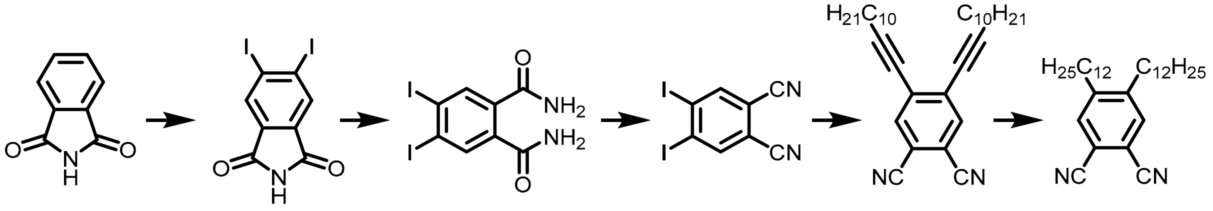

13 **14** **15** **16** **17** **5**

Scheme S2: Synthesis of 4,5-bis(dodecyl)phthalonitrile, **5**, from phthalimide, **13**.

4,5-Diiodophthalimide, ***14.*** Phthalimide (10 g, 68 mmol), iodine (24 g, 189 mmol) and 20% fuming sulphuric acid (70 ml) was stirred at 70 °C for 24 hours, after which the reaction mixture was poured onto crushed ice (*ca* 400 ml). The resulting solids were filtered, washed with water (100 ml), 2 % K_2CO_3 (100 ml), saturated Na_2SO_3 (100 ml) and then water again (100 ml). Recrystallization from acetone/water yielded **14** as light-yellow crystals (12 g, 30 mmol, 44 % yield). ATR FTIR/cm^{-1}: υ(C=O) 1721, υ(N-H) 3201 – 3402. ^{1}H NMR (DMSO-*d*$_6$, 400 MHz)/ppm: 11.44 (s, 1H), 8.26 (s, 2H).

4,5-Diiodobenzene-1,2-dicarboxamide, ***15.*** 4,5-Diiodophthalimide (1.3 g, 3.3 mmol) was stirred with aqueous ammonium (25%, 130 ml) for 90 minutes at 60 °C. The cooled mixture was filtered, and the solids washed with water (50 ml) and cold methanol (50 ml). Drying at 50 mmHg (room temperature) for 1 hour yielded **15** as an off-white solid (1.06 g, 2.35 mmol, 71 % yield). ATR FTIR/cm^{-1}: υ(C=O) 1693, υ(N-H) 3164 – 3427. ^{1}H NMR (DMSO-*d*$_6$, 400 MHz)/ppm: 7.93 (s, 2H), 7.83 (s, 2H) 7.42 (s, 2H).

4,5-Diiodophthalonitrile, ***16.*** 4,5-Diiodo-1,2-dicarboxamide (0.5 g, 1.2 mmol) in a mixture of anhydrous pyridine/dioxane (5:2, 28 ml) was pre-cooled to 0 °C in an ice bath under argon. $POCl_3$ (1.2 ml, 2.0 g, 13 mmol) was added to the stirring mixture dropwise over a period of *ca* 15 minutes, after which the ice bath was removed, and the reaction mixture was allowed to continue stirring under argon overnight. The mixture was then poured onto ice (*ca* 100 ml) and extracted with ethyl acetate (3 x 25 ml). Water washing (2 x 100 ml) of the combined

organic layer, followed by drying ($MgSO_4$) and solvent removal afforded a dark-brown residue which was recrystallized from methanol to yield **16** as light-yellow, needle-like crystals (0.24 g, 0.63 mmol, 53 % yield). ATR FTIR/cm^{-1}: ʋ(C≡N) 2231, ʋ(C-H) 3033. ^{1}H NMR ($CDCl_3$, 400 MHz)/ppm: 8.22 (s, 2H).

4,5-Bis(dodecyne)phthalonitrile, ***17.*** 4,5-Diiodophthalonitrile (2.8 g, 7.4 mmol) was dissolved in a mixture of triethylamine (40 ml) and anhydrous DMF (4 ml). Argon was bubbled through the reaction mixture for 15 minutes prior to the addition of $Pd(PPh_3)_2Cl_2$ (300 mg). The mixture was then heated to 100 °C, after which 1-dodecyne (3.9 ml, 3.07 g, 18.5 mmol) was added dropwise over a period of *ca* 15 minutes. Refluxing at 100 °C was maintained for 2 hours, followed by cooling and filtration. The solid residue was washed with diethyl ether until the filtrate was colourless, after which the solvent was removed under reduced pressure and the residue subjected to column chromatography over silica using 10 % diethyl ether in hexanes as eluent. After solvent removal, the first band was recrystallized from hexanes to yield **17** as an off-white waxy residue (2.4 g, 5.25 mmol, 71 % yield). ATR FTIR/cm^{-1}: ʋ(C≡N) 2229, ʋ(C-H) 2850, ʋ(C-H) 2955. ^{1}H NMR ($CDCl_3$, 600 MHz)/ppm: 7.73 (s, 2H), 2.49 (t, 4H, J =7.11 Hz), 1.63 (m, 4H, J = 7.4 Hz), 1.46 (m, 4H, J = 5.9 Hz), 1.28 (s, 32 H), 0.88 (t, 6H, J = 7.03 Hz).

4,5-Bis(dodecyl)phthalonitrile, ***5.*** 4,5-Bis(dodecyne)phthalonitrile (1.0 g, 2.2 mmol), palladium on carbon (10 % wt., 100 mg) and anhydrous THF (30 ml) was stirred under an initial hydrogen gas pressure of 6 bar for 2.75 hours at room temperature. The mixture was then passed through a short silica column (5 g) using THF as eluent and the solvent was removed. After a second chromatographic separation (silica, 10 % diethyl ether in hexanes) and solvent removal, the remaining residue was crystallized from hexanes to yield **5** as off-white crystals (920 mg, 2 mmol, 90 % yield). ATR FTIR/cm^{-1}: ʋ(C≡N) 2229, ʋ(C-H) 2847,

υ(C-H) 2955. ^{1}H NMR ($CDCl_3$, 600 MHz)/ppm: 7.55 (s, 2H), 2.67 (t, 4H, *J* = 8.0 Hz), 1.57 (m, 4H, *J* = 7.6 Hz), 1.26 (s, 36 H), 0.88 (t, 6H, *J* = 7.0 Hz).

B. ^{1}H NMR SPECTRA

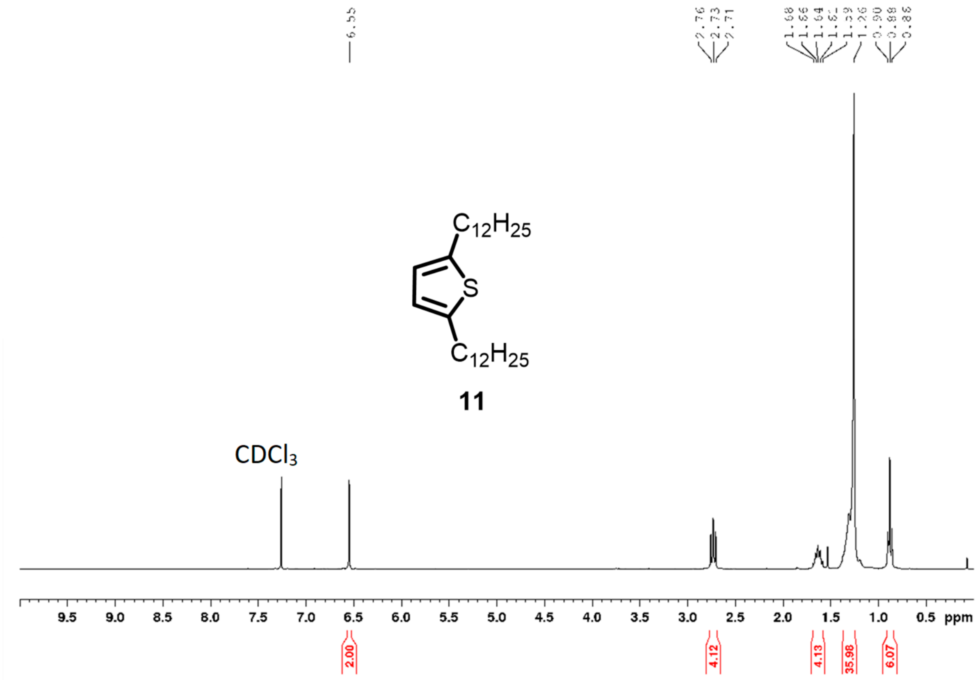

Figure S1: ^{1}H NMR spectrum of 2,5-bis(dodecyl)thiophene, **11**.

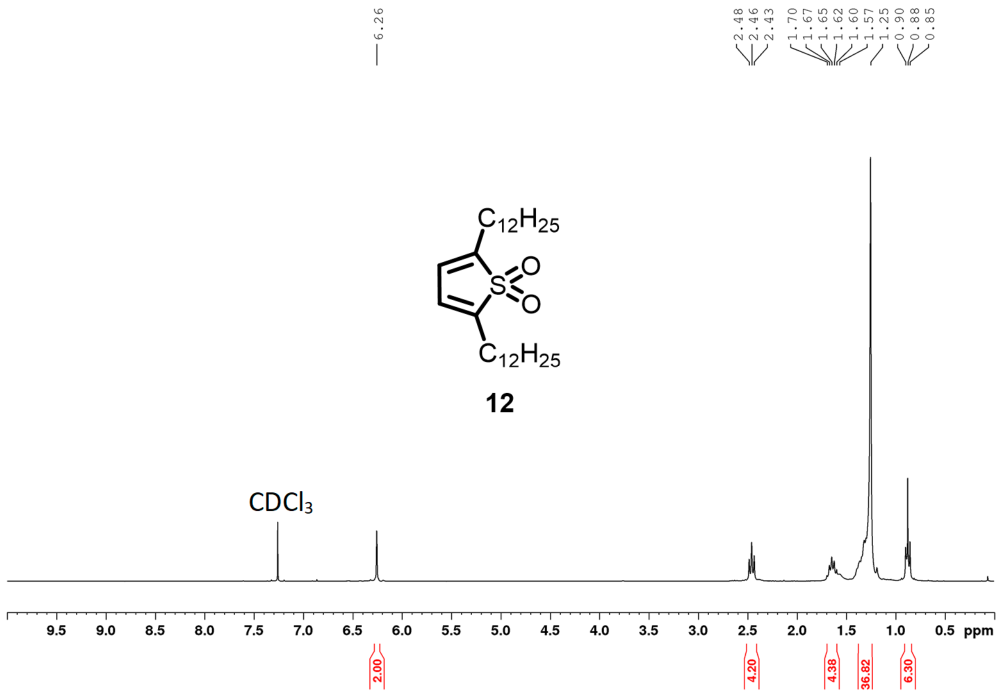

Figure S2: ^{1}H NMR spectrum of 2,5-bis(dodecyl)thiopehen-1,1-dioxide, **12**.

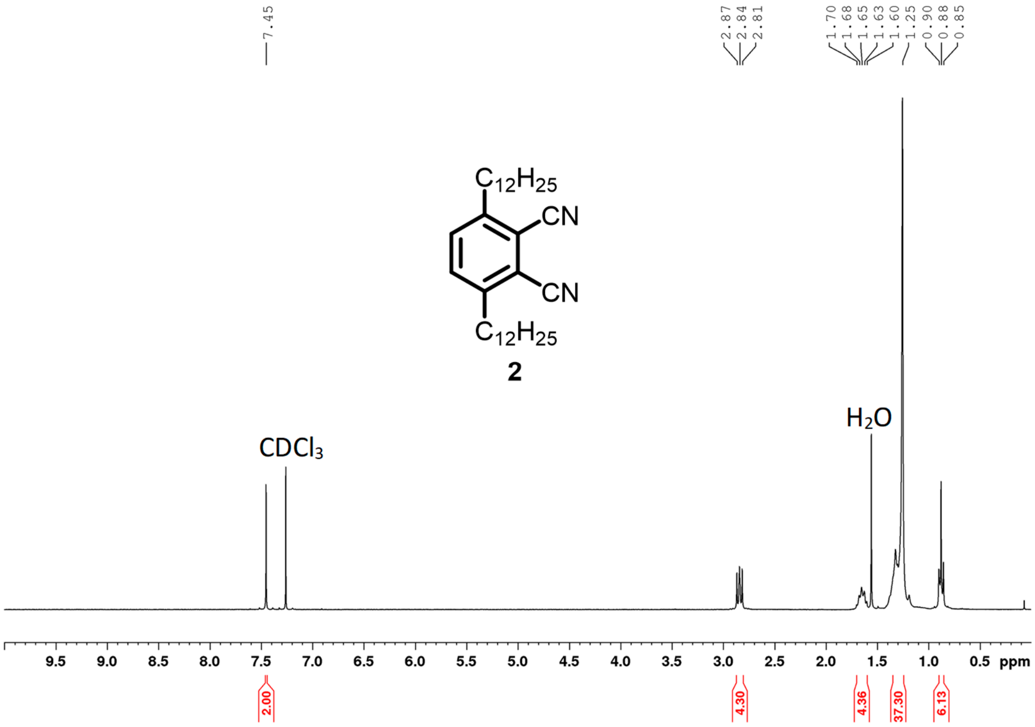

Figure S3: ^{1}H NMR spectrum of 3,6-bis(dodecyl)phthalonitrile, **2**.

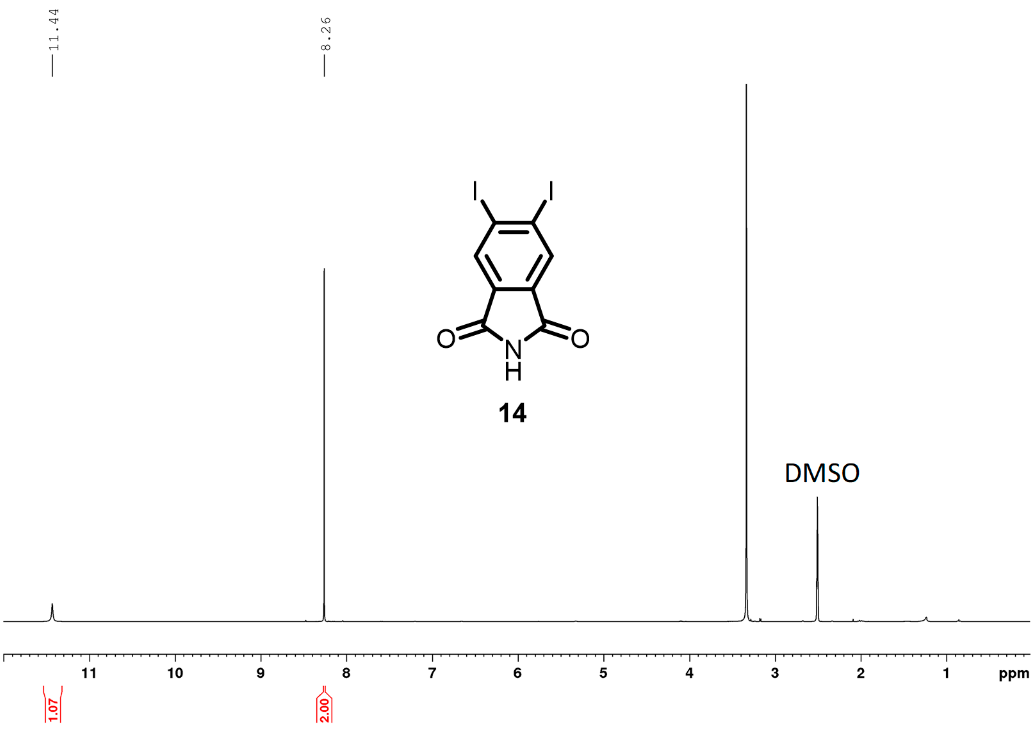

Figure S4: ^{1}H NMR spectrum of 4,5-diiodophthalimide, **14**.

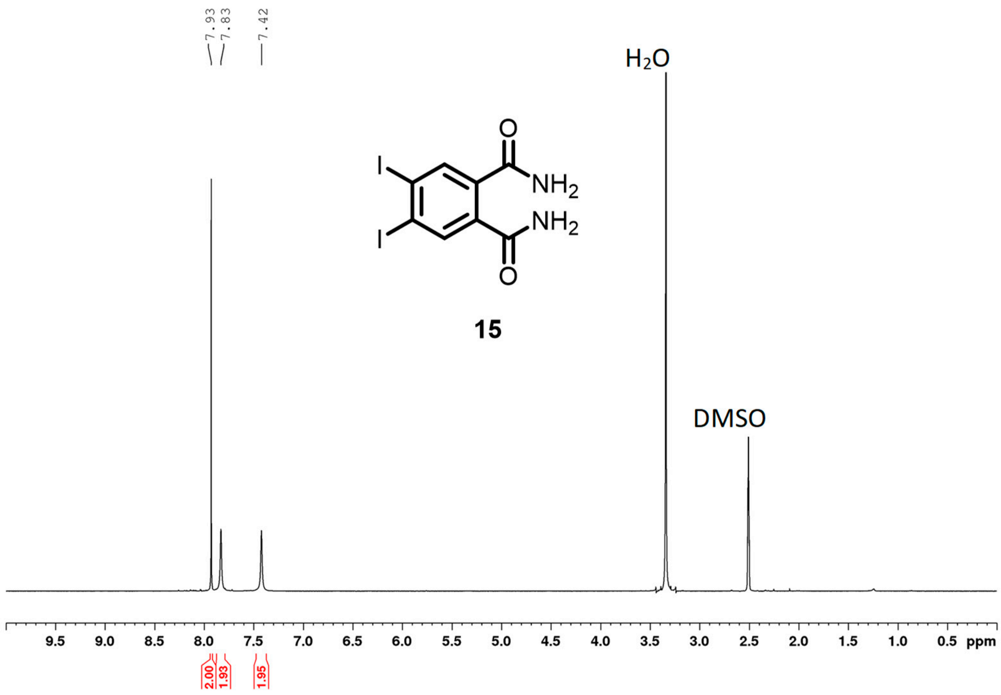

Figure S5: ^{1}H NMR spectrum of 4,5-diiodobenzene-1,2-carboxamide, **15**.

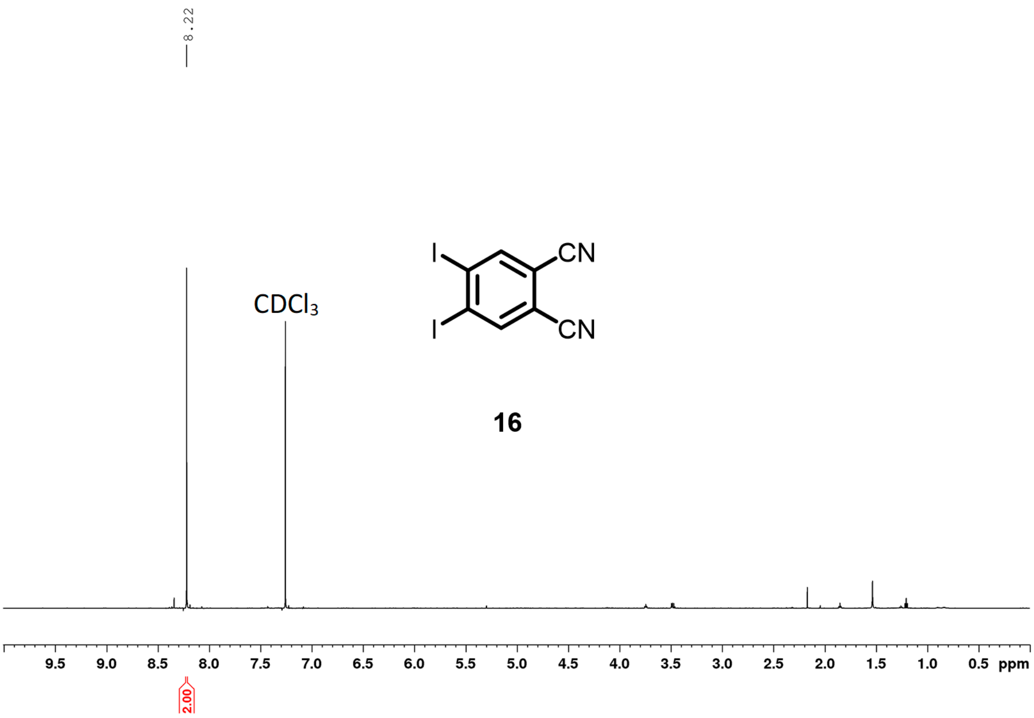

Figure S6: ¹H NMR spectrum of 4,5-diiodophthalonitrile, **16**.

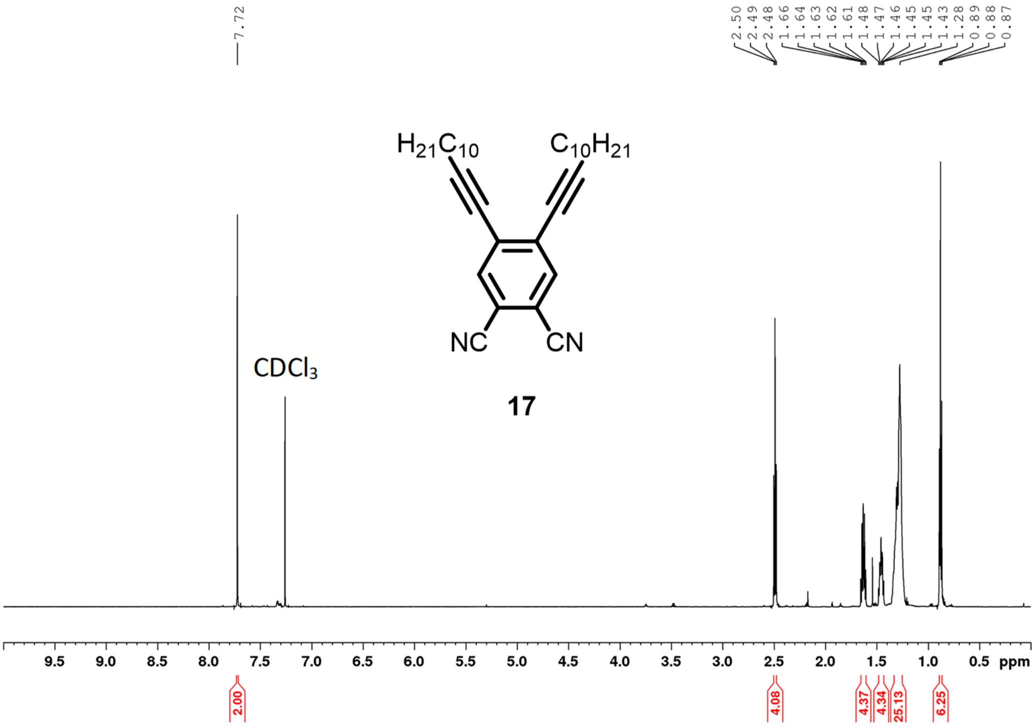

Figure S7: ¹H NMR spectrum of 4,5-bis(dodecyne)phthalonitrile, **17**.

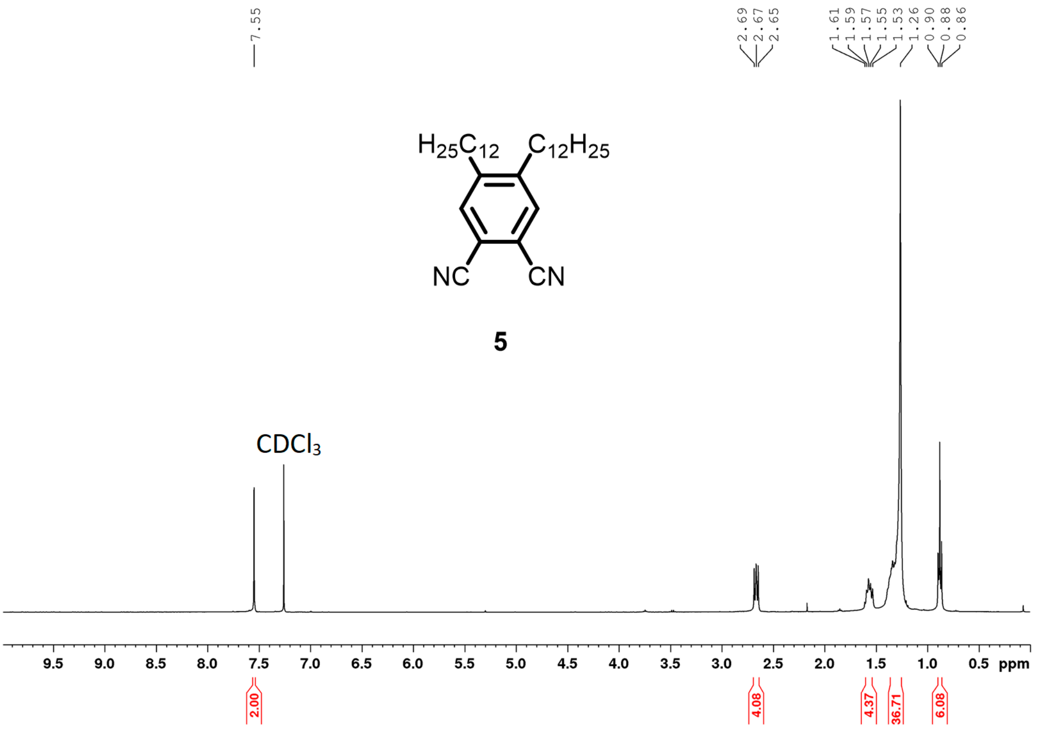

Figure S8: ^{1}H NMR spectrum of 4,5-bis(dodecyl)phthalonitrile, **5.**

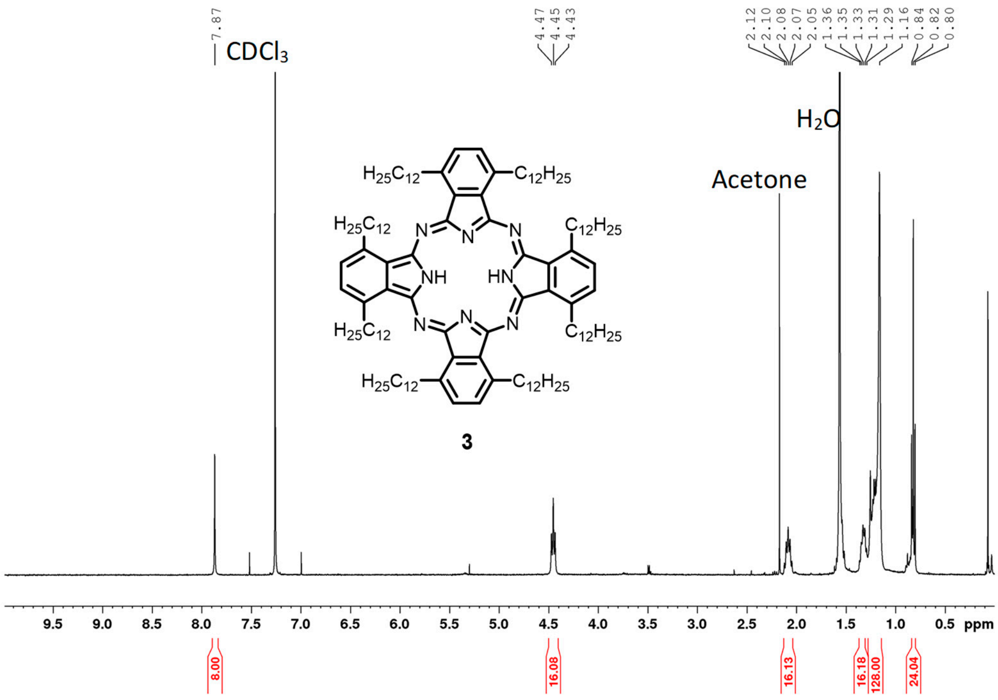

Figure S9: ^{1}H NMR spectra of 1,4,8,11,15,18,22,25-octakis(dodecyl)phthalocyanine, **3**.

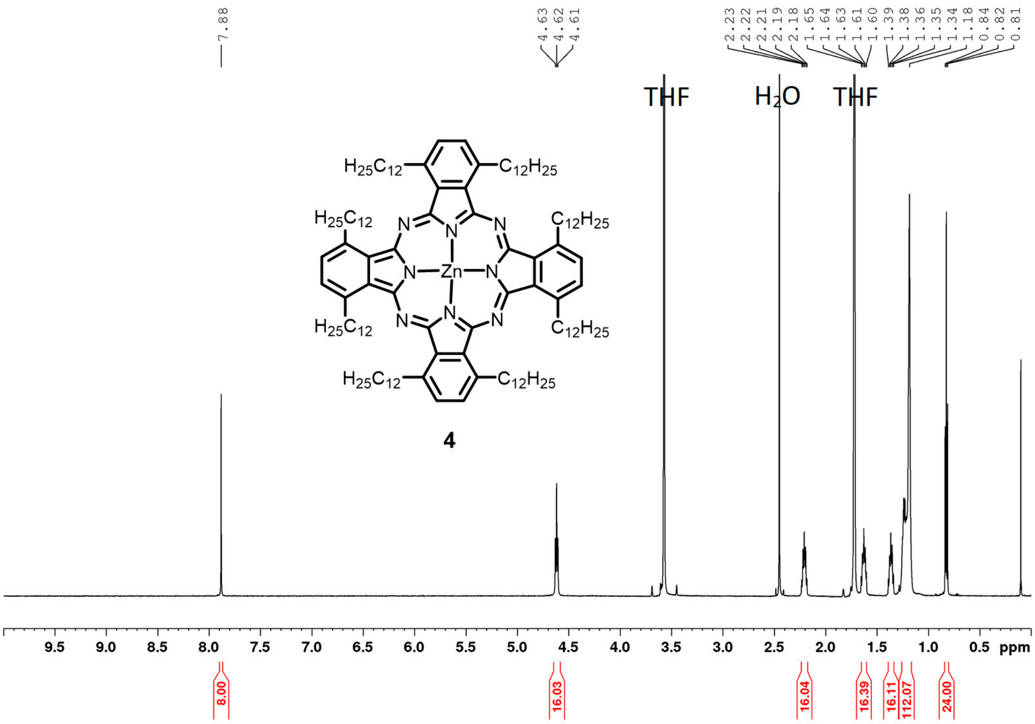

Figure S10: ^{1}H NMR of 1,4,8,11,15,18,22,25-octakis(dodecyl)phthalocyaninatozinc(II), **4**.

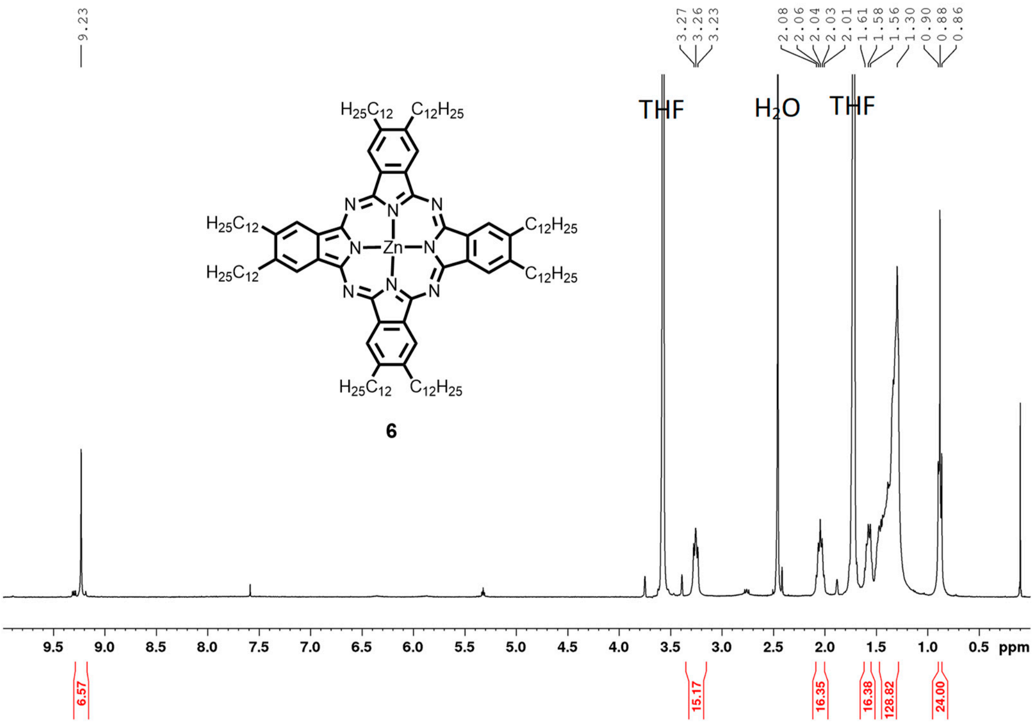

Figure S11: ^{1}H NMR of 2,3,9,10,16,17,23,24-octakis(dodecyl)phthalocyaninatozinc(II), **6**.

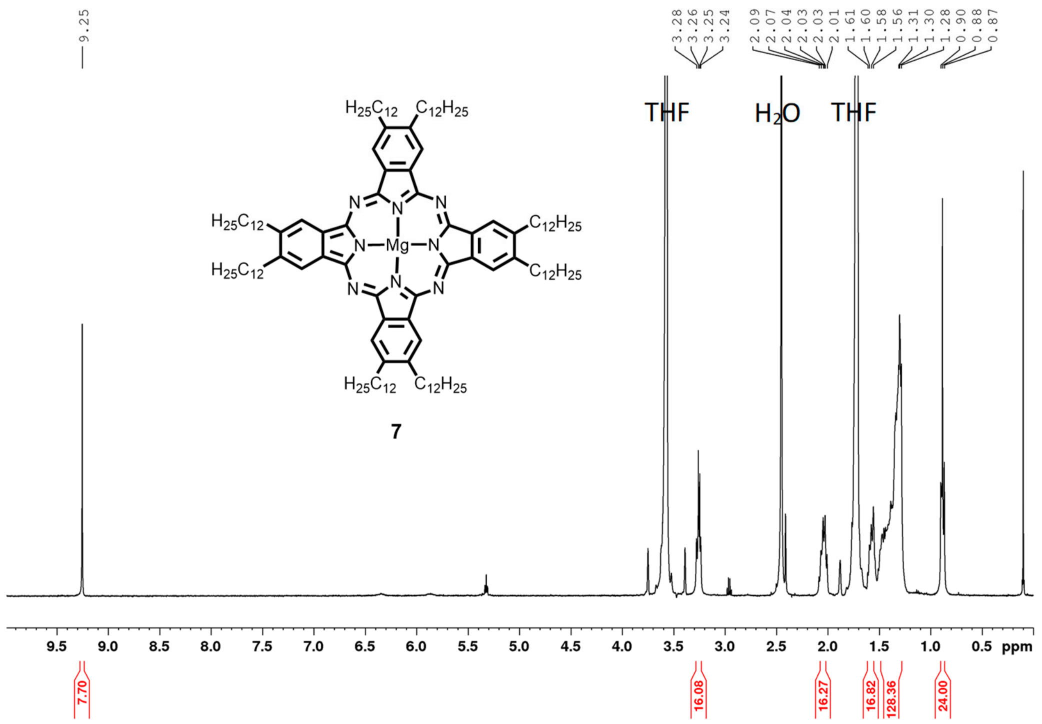

Figure S12: ^{1}H NMR of 2,3,9,10,16,17,23,24-octakis(dodecyl)phthalocyaninatomagnesium(II), **7**.

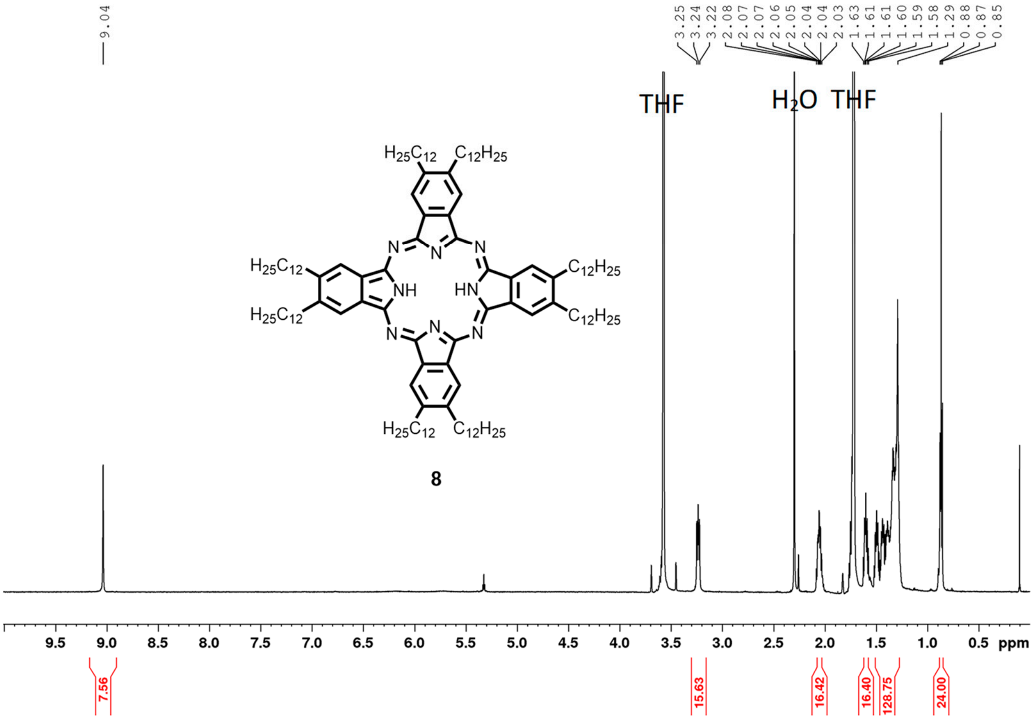

Figure S13: ^{1}H NMR of 2,3,9,10,16,17,23,24-octakis(dodecyl)phthalocyanine, **8**.

C. FOURIER TRANSFORM INFRA-RED ATTENUATED TOTAL REFLECTANCE SPECTRA

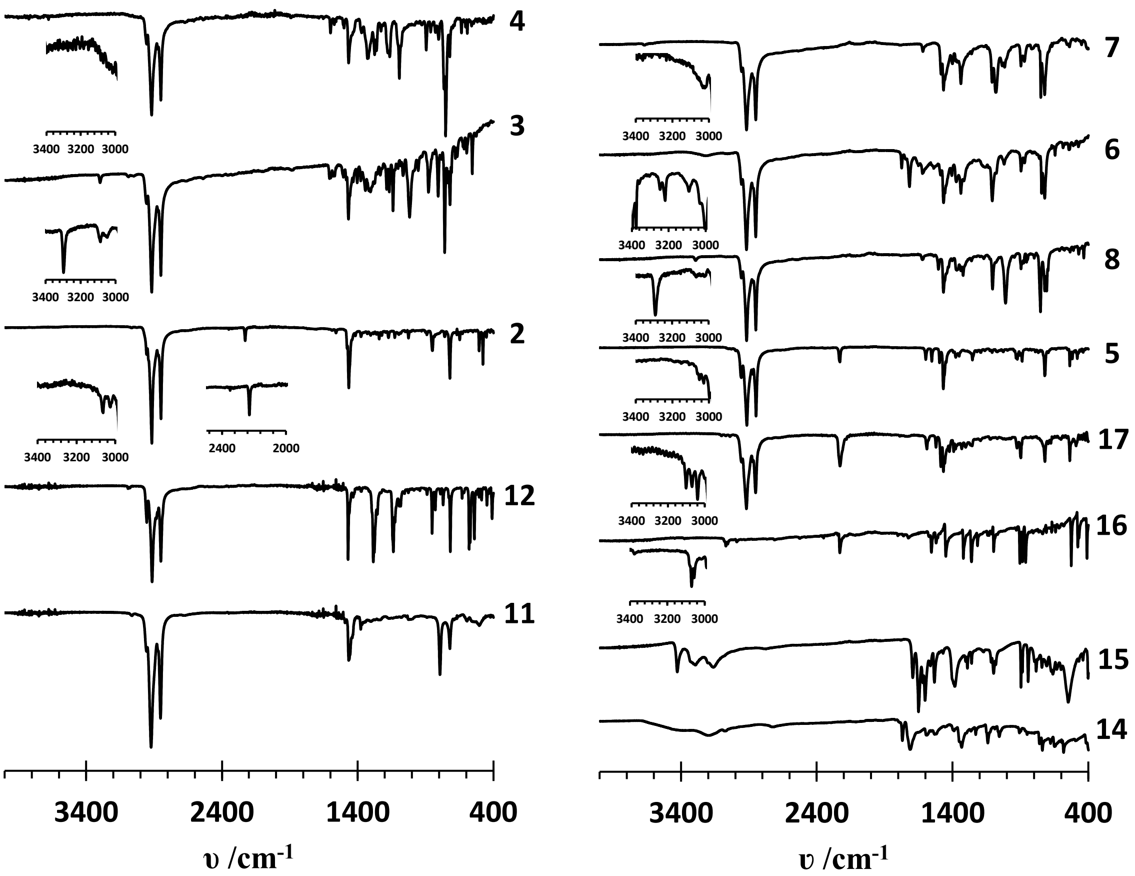

Figure S14: Left: IR spectra for non-peripherally substituted phthalocyanines and related precursors. Right: IR spectra of peripherally substituted phthalocyanines and related precursors. Compound numbers are indicated next to each spectrum.

D. UV-VIS SPECTROSCOPY

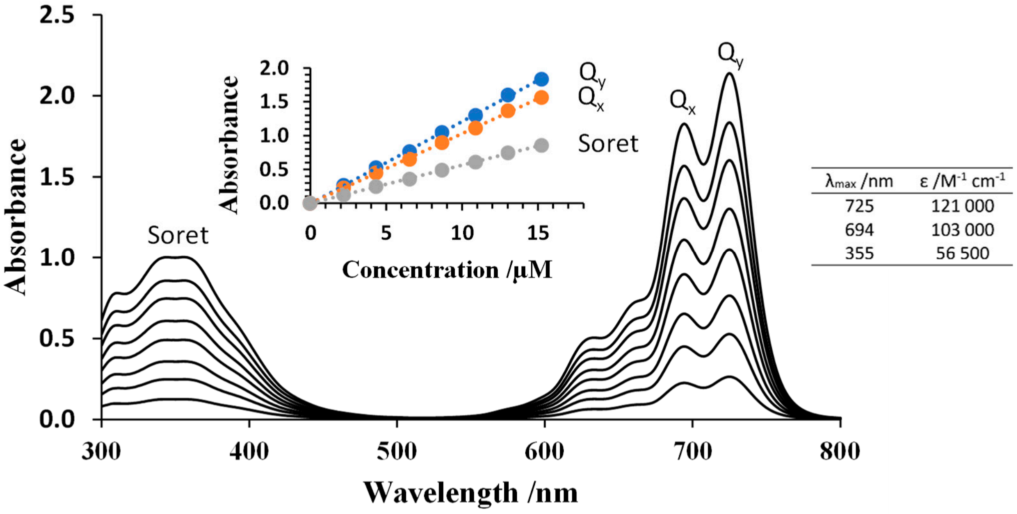

λ_{max} /nm	ε /M^{-1} cm^{-1}
725	121 000
694	103 000
355	56 500

Figure S15: UV-vis spectra of **3** recorded in neat THF at 25 ºC. Insertion: Beer-Lambert law is followed up to concentrations of 15 µM.

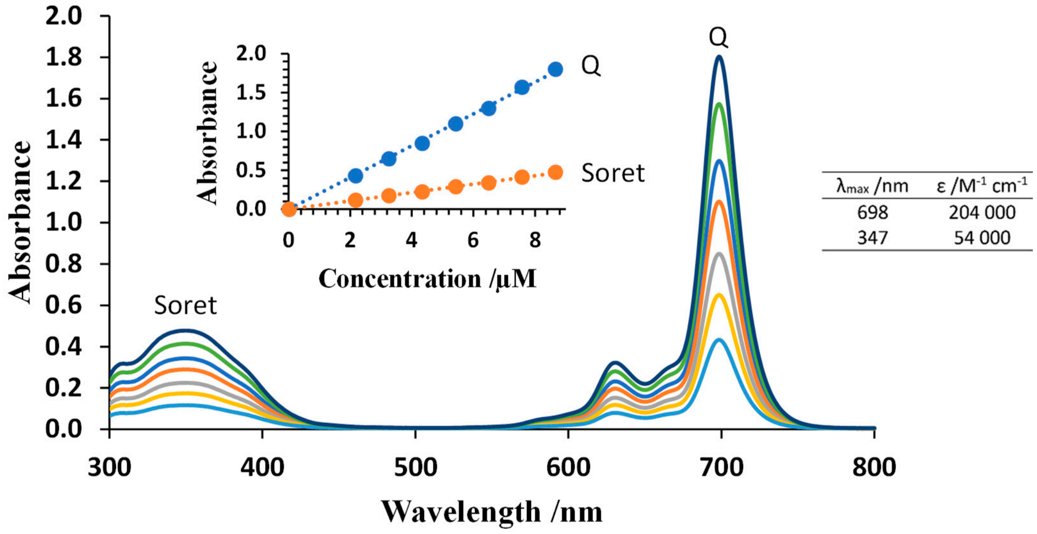

λ_{max} /nm	ε /M^{-1} cm^{-1}
698	204 000
347	54 000

Figure S16: UV-vis spectra of **4** recorded in THF at 25 ºC. Insertion: Beer-Lambert law is followed up to concentrations of 9 µM**.**

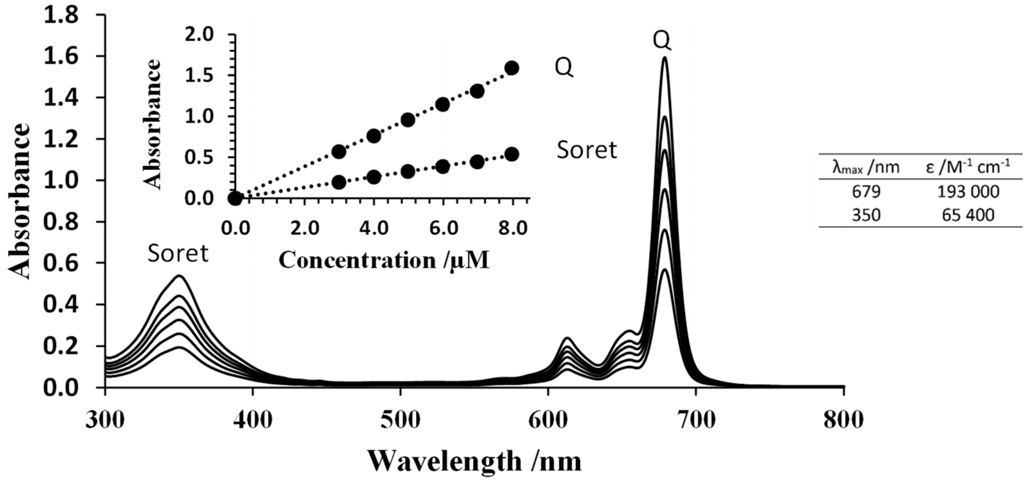

Figure S17: UV-vis spectra of **6** recorded in THF at 25 ºC. Insertion: Beer-Lambert law is followed up to concentrations of 8 µM.

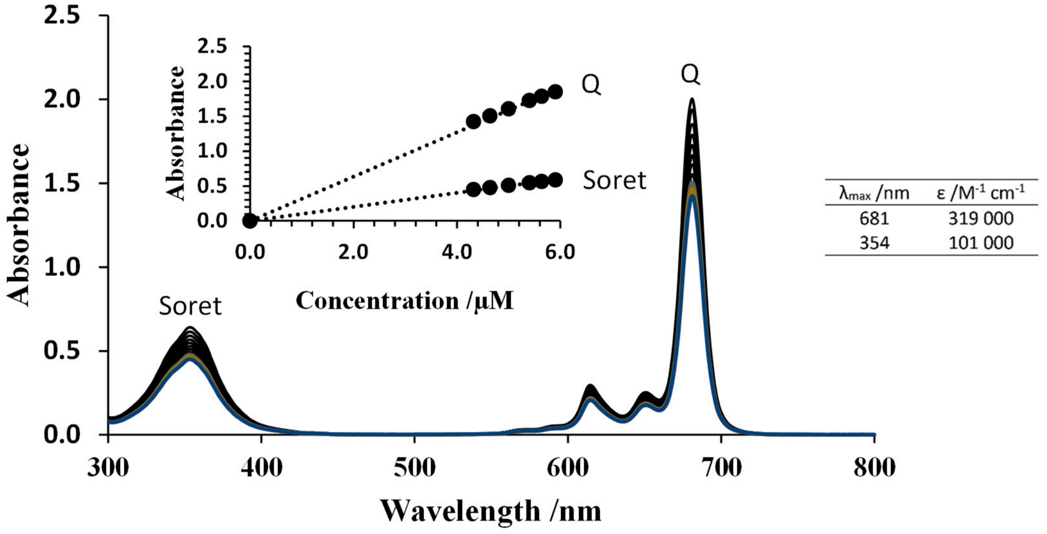

Figure S18: UV-vis spectra of **7** recorded in THF at 25 ºC. Insertion: Beer-Lambert law is followed up to concentrations of 6 µM.

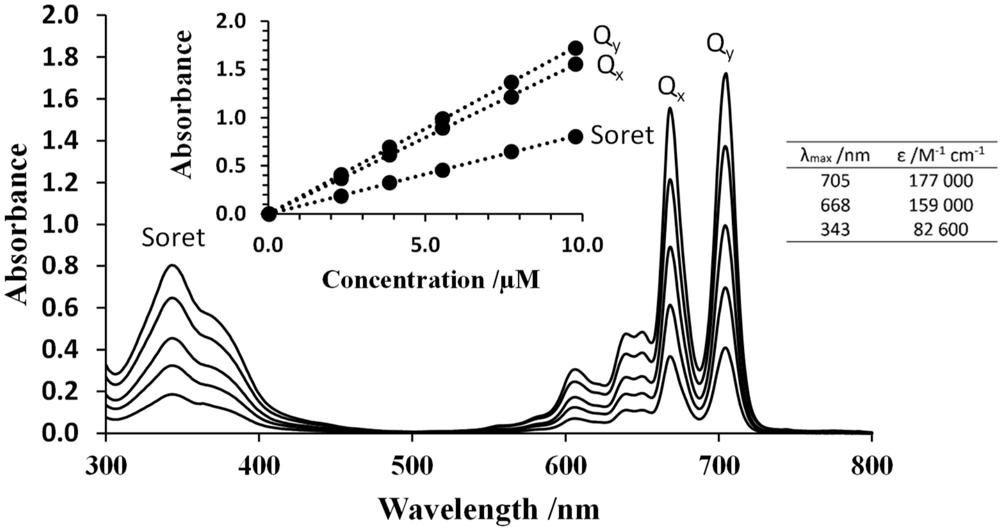

Figure S19: UV-vis spectra of **8** recorded in THF at 60 ºC. Insertion: Beer-Lambert law is followed up to concentrations of *10* µM.

E. ELECTROCHEMISTRY

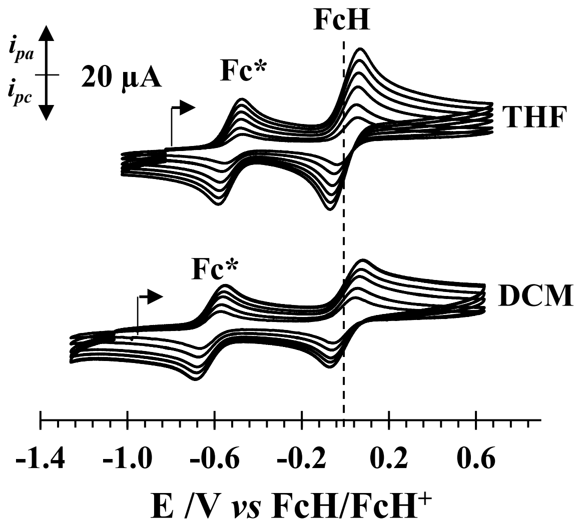

Figure S20: CV's of decamethylferrocene *vs*. ferrocene in DCM (bottom) and THF (top). CV's were recorded 25 °C.

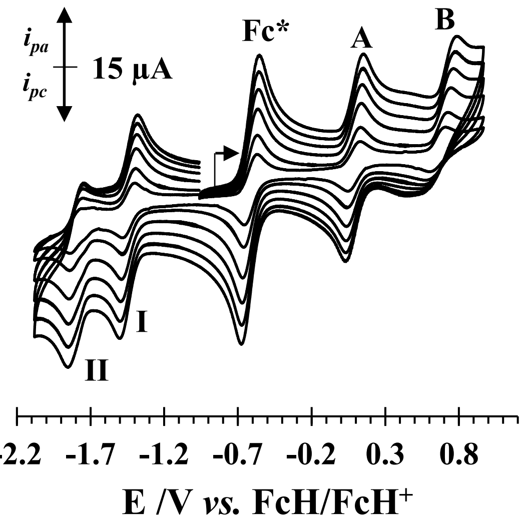

Figure S21: The 50, 100, 200, 300, 400 and 500 mV/s scan rate cyclic voltammograms for **3** recorded in DCM at 25 ºC.

Table S1: Peak anodic potentials, E_{pa}, peak potential differences, ΔE_p, formal reduction potentials, $E^{o\prime}$, peak anodic or cathodic currents, i_{pa} and i_{pc} respectively, and current ratio, i_{pc}/i_{pa} or i_{pa}/i_{pc} for the 50, 100, 200, 300, 400 and 500 mV/s scan rate of **3** in DCM recorded at 25 °C.

υ /mV.s^{-1}	E_{pa}/V	ΔE_p/mV^a	$E^{o\prime}$/V	i_{pa}/μA	i_{pc}/i_{pa}	υ /mV.s^{-1}	E_{pa}/V	ΔE_p/mV^a	$E^{o\prime}$/V	i_{pc}/μA	i_{pa}/i_{pc}
**Wave A**						**Wave I**					
**50**	0.134	87	0.091	3.11	0.97	**50**	-1.400	88	-1.444	2.67	0.80
**100**	0.136	97	0.088	4.75	1.00	**100**	-1.396	93	-1.443	4.25	0.93
**200**	0.139	101	0.089	6.83	0.98	**200**	-1.390	101	-1.441	5.67	0.91
**300**	0.141	105	0.089	8.17	0.98	**300**	-1.389	105	-1.442	7.00	0.79
**400**	0.145	115	0.088	9.58	0.98	**400**	-1.381	118	-1.440	8.13	0.85
**500**	0.148	124	0.086	10.45	1.00	**500**	-1.379	123	-1.441	9.09	0.75
**Wave B**						**Wave II**					
**50**	0.710	116	0.652	2.49	0.64	**50**	-1.758	84	-1.800	2.13	0.96
**100**	0.726	143	0.655^b	3.38	0.56	**100**	-1.754	92	-1.800	3.13	0.96
**200**	0.743	183	0.652^b	4.83	0.45	**200**	-1.750	100	-1.800	4.67	1.00
**300**	0.754	211	0.649^b	5.33	0.53	**300**	-1.749	105	-1.801	5.33	0.97
**400**	0.770	250	0.645^b	5.83	0.54	**400**	-1.748	110	-1.803	6.25	0.97
**500**	0.781	277	0.643^b	6.82	0.47	**500**	-1.742	118	-1.801	6.82	1.00

$^a\Delta E_p = E_{pa} - E_{pc}$. ^{b}Strictly speaking, the Nernst equation and equations derived therefrom only hold true for electrochemically reversible processes, i. e. those that exhibit ΔE_p approaching 59 mV.

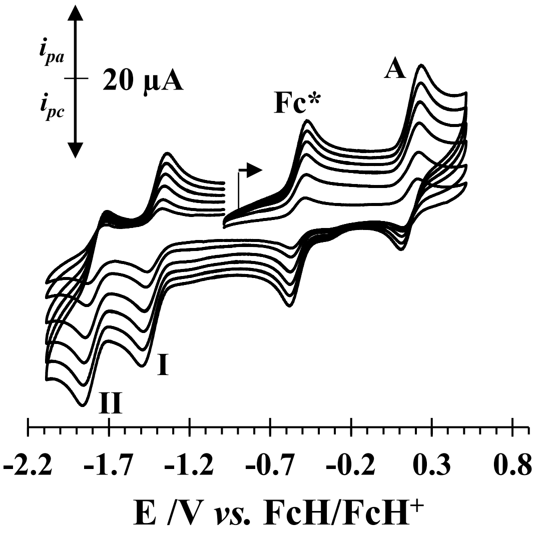

Figure S22: The 50, 100, 200, 300, 400 and 500 mV/s scan rate cyclic voltammograms for **3** recorded in THF at 25 ºC.

Table S2: Peak anodic potentials, E_{pa}, peak potential differences, ΔE_p, formal reduction potentials, $E^{o\prime}$, peak anodic or cathodic currents, i_{pa} and i_{pc} respectively, and current ratio, i_{pc}/i_{pa} or i_{pa}/i_{pc} for the 50, 100, 200, 300, 400 and 500 mV/s scan rate of **3** in THF recorded at 25 °C.

υ /mV.s⁻¹	E_{pa}/V	ΔE_p/mV[a]	$E^{o\prime}$/V	i_{pa}/μA	i_{pc}/i_{pa}	υ /mV.s⁻¹	E_{pa}/V	ΔE_p/mV[a]	$E^{o\prime}$/V	i_{pc}/μA	i_{pa}/i_{pc}
**Wave A**						**Wave I**					
**50**	0.210	87	0.167	2.28	0.86	**50**	-1.371	92	-1.417	1.95	1.00
**100**	0.221	99	0.172	3.40	0.9	**100**	-1.364	107	-1.418	3.40	0.88
**200**	0.225	110	0.170	4.71	0.75	**200**	-1.355	124	-1.417	4.12	0.86
**300**	0.227	114	0.170	6.67	0.80	**300**	-1.351	130	-1.416	5.33	0.88
**400**	0.230	122	0.169	7.50	0.80	**400**	-1.343	143	-1.415[b]	6.00	1.00
**500**	0.233	127	0.170	8.33	0.80	**500**	-1.337	160	-1.417[b]	6.67	0.87
						**Wave II**					
						**50**	-1.718	114	-1.775	2.60	0.88
						**100**	-1.716	121	-1.777	3.40	0.88
						**200**	-1.708	140	-1.778[b]	4.12	0.86
						**300**	-1.704	149	-1.779[b]	5.33	0.88
						**400**	-1.703	153	-1.780[b]	6.00	0.88
						**500**	-1.696	167	-1.780[b]	6.67	0.87

[a] $\Delta E_p = E_{pa} - E_{pc}$. [b] Strictly speaking, the Nernst equation and equations derived therefrom only hold true for electrochemically reversible processes, i. e. those that exhibit ΔE_p approaching 59 mV.

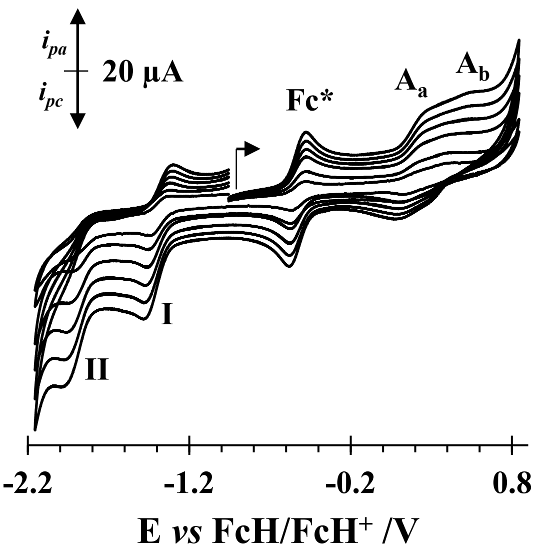

Figure S23: The 50, 100, 200, 300, 400 and 500 mV/s scan rate cyclic voltammograms for **8** recorded in THF at 60 ºC.

Table S3: Peak anodic potentials, E_{pa}, peak potential differences, ΔE_p, formal reduction potentials, $E^{o\prime}$, peak anodic or cathodic currents, i_{pa} and i_{pc} respectively, and current ratio, i_{pc}/i_{pa} or i_{pa}/i_{pc} for the 50, 100, 200, 300, 400 and 500 mV/s scan rate of **8** in THF recorded at 60 °C.

υ /mV.s^{-1}	E_{pa}/V	ΔE_p/mV^a	$E^{o\prime}$/V	i_{pa}/μA	i_{pc}/i_{pa}	υ /mV.s^{-1}	E_{pa}/V	ΔE_p/mV^a	$E^{o\prime}$/V	i_{pc}/μA	i_{pa}/i_{pc}
**Wave A_a**						**Wave I**					
**50**	0.204	83	0.163	1.50	0.60	**50**	-1.336	104	-1.388	2.44	0.89
**100**	0.227	111	0.172	1.56	0.80	**100**	-1.326	127	-1.390	3.44	0.82
**200**	0.243	13	0.178	2.50	1.00	**200**	-1.318	144	-1.390^b	5.00	0.83
**300**	0.247	136	0.179	3.53	0.83	**300**	-1.305	169	-1.390^b	6.47	0.91
**400**	0.252	149	0.178^b	3.75	0.83	**400**	-1.298	183	-1.390^b	7.50	0.83
**500**	0.262	161	0.182^b	3.91	0.67	**500**	-1.293	191	-1.389^b	8.48	0.92
**Wave A_b**						**Wave II**					
**50**	0.409	83	0.368	1.20	0.75	**50**	-1.797	119	-1.857	1.63	1.00
**100**	0.416	98	0.367	1.80	0.83	**100**	-1.785	135	-1.853	2.50	1.00
**200**	0.427	111	0.372	2.00	0.88	**200**	-1.781	148	-1.855^b	4.17	0.90
**300**	0.432	119	0.373	2.25	0.89	**300**	-1.778	169	-1.863^b	3.53	0.83
**400**	0.54	230	0.425^b	3.04	0.89	**400**	-1.773	187	-1.867^b	5.00	0.75
**500**	0.546	242	0.425^b	3.38	0.90	**500**	-1.767	207	-1.871^b	5.20	1.00

[a] $\Delta E_p = E_{pa} - E_{pc}$. [b] Strictly speaking, the Nernst equation and equations derived therefrom only hold true for electrochemically reversible processes, i. e. those that exhibit ΔE_p approaching 59 mV.

Table S4: Peak anodic potentials, E_{pa}, peak potential differences, ΔE_p, formal reduction potentials, $E^{o\prime}$, peak anodic or cathodic currents, i_{pa} and i_{pc} respectively, and current ratio, i_{pc}/i_{pa} or i_{pa}/i_{pc} for the 50, 100, 200, 300, 400 and 500 mV/s scan rate of **6** in THF recorded at 25 °C.

υ /mV.s^{-1}	E_{pa}/V	ΔE_p/mV[a]	$E^{o\prime}$/V	i_{pa}/μA	i_{pc}/i_{pa}	υ /mV.s^{-1}	E_{pa}/V	ΔE_p/mV[a]	$E^{o\prime}$/V	i_{pc}/μA	i_{pa}/i_{pc}
**Wave A_a**						**Wave I**					
**50**	0.065	81	0.024	1.79	0.80	**50**	-1.581	106	-1.634	2.50	0.78
**100**	0.067	86	0.024	2.68	0.93	**100**	-1.578	113	-1.633	3.57	0.85
**200**	0.073	101	0.022	2.38	0.91	**200**	-1.575	120	-1.635	4.29	0.88
**300**	0.077	110	0.022	2.62	0.79	**300**	-1.571	129	-1.635	5.48	0.96
**400**	0.084	121	0.023	3.55	0.85	**400**	-1.564	137	-1.632	7.10	0.95
**500**	0.086	125	0.023	3.87	0.75	**500**	-1.561	142	-1.632[b]	8.25	0.94
**Wave A_b**											
**50**	0.181	74	0.144	1.61	0.78						
**100**	0.181	78	0.142	1.79	0.80						
**200**	0.184	84	0.142	2.14	1.00						
**300**	0.186	88	0.142	2.86	0.92						
**400**	0.193	98	0.144	3.87	0.83						
**500**	0.193	103	0.141	3.55	1.00						

[a]$\Delta E_p = E_{pa} - E_{pc}$. [b]Strictly speaking, the Nernst equation and equations derived therefrom only hold true for electrochemically reversible processes, i. e. those that exhibit ΔE_p approaching 59 mV.

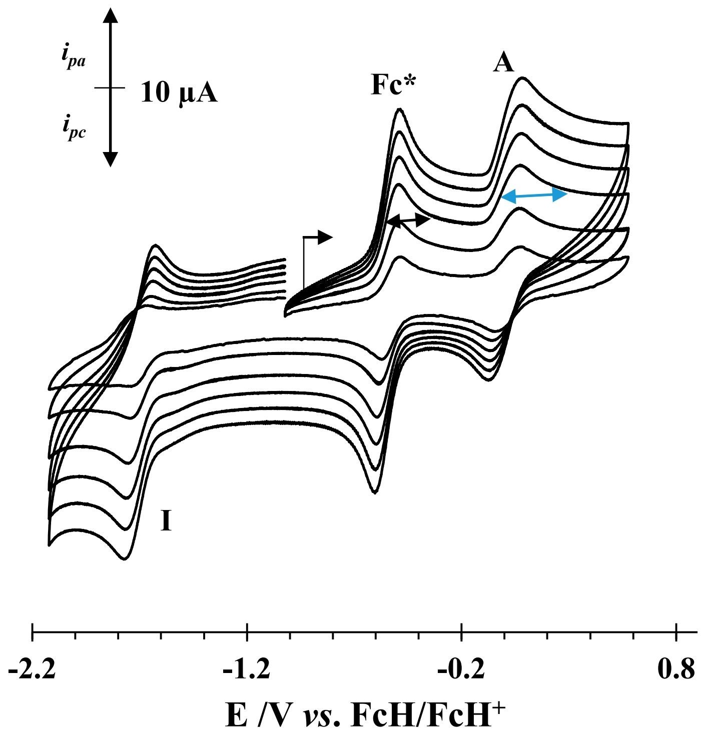

Figure S24: The 50, 100, 200, 300, 400 and 500 mV/s scan rate cyclic voltammograms for **7** recorded in THF at 25 ºC. Peak widths at half peak heights at a scan rate of 200 mV/s are

illustrated for the ideal anodic half-cycle of decamethylferrocene and for the anodic half-cycle of wave A. The former is 196 mV wide, while the latter is 317 mV. This enlarged peak width at half height of **7** is also consistent with dimerizing, just as **6** and **8**, but the resolution between waves A_a and wave A_b is so poor that no peak potentials nor peak currents can be assigned to these two wave components. Instead, only the observed peak potentials and currents are reported in Table S5 and also in the main manuscript in Table 3.

Table S5: Peak anodic potential, E_{pa}, peak potential difference, ΔE_p, formal reduction potential, $E^{o\prime}$, peak current in the forward scanning direction, i_f, and current ratio, i_f/i_b, of each labelled redox couple for the 50, 100, 200, 300, 400 and 500 mV/s scan rate CV of 7 in THF.

υ /mV.s^{-1}	E_{pa}/V	ΔE_p/mV[a]	$E^{o\prime}$/V	i_{pa}/µA	i_{pc}/i_{pa}	υ /mV.s^{-1}	E_{pa}/V	ΔE_p/mV[a]	$E^{o\prime}$/V	i_{pc}/µA	i_{pa}/i_{pc}
**Wave A**						**Wave I**					
**50**	0.076	122	0.014	1.60	0.97	**50**	-1.663	77	-1.701	1.30	0.92
**100**	0.080	135	0.012	2.40	0.96	**100**	-1.646	95	-1.693	1.90	0.95
**200**	0.079	143	0.007	3.20	0.94	**200**	-1.639	115	-1.696	3.60	0.92
**300**	0.083	151	0.007[b]	3.80	0.90	**300**	-1.636	131	-1.701	4.00	0.90
**400**	0.087	155	0.009[b]	4.20	0.84	**400**	-1.631	137	-1.699	5.00	0.88
**500**	0.080	155	0.002[b]	5.00	0.86	**500**	-1.626	149	-1.700[b]	5.60	0.89

[a]$\Delta E_p = E_{pa} - E_{pc}$. [b]Strictly speaking, the Nernst equation and equations derived therefrom only hold true for electrochemically reversible processes, i. e. those that exhibit ΔE_p approaching 59 mV.

Table S6: Peak anodic potentials, E_{pa}, peak potential differences, ΔE_p, formal reduction potentials, $E^{o\prime}$, peak anodic or cathodic currents, i_{pa} and i_{pc} respectively, and current ratio, i_{pc}/i_{pa} or i_{pa}/i_{pc} for the 50, 100, 200, 300, 400 and 500 mV/s scan rate of **4** in THF recorded at 25 °C.

υ /mV.s^{-1}	E_{pa}/V	ΔE_p/mV[a]	$E^{o\prime}$/V	i_{pa}/µA	i_{pc}/i_{pa}	υ /mV.s^{-1}	E_{pa}/V	ΔE_p/mV[a]	$E^{o\prime}$/V	i_{pc}/µA	i_{pa}/i_{pc}
**Wave A**						**Wave I**					
**50**	0.102	93	0.056	2.90	0.97	**50**	-1.561	120	-1.621	2.00	1.00
**100**	0.105	105	0.053	3.60	0.97	**100**	-1.556	128	-1.620	2.60	1.00
**200**	0.112	111	0.056	5.04	0.96	**200**	-1.544	164	-1.626[b]	3.60	1.00
**300**	0.112	117	0.054	6.20	0.97	**300**	-1.536	177	-1.625[b]	4.40	1.00
**400**	0.113	122	0.052	7.10	0.96	**400**	-1.529	199	-1.629[b]	4.80	1.00
**500**	0.122	137	0.054	8.50	0.95	**500**	-1.524	216	-1.632[b]	5.00	1.00

[a]$\Delta E_p = E_{pa} - E_{pc}$. [b]Strictly speaking, the Nernst equation and equations derived therefrom only hold true for electrochemically reversible processes, i. e. those that exhibit ΔE_p approaching 59 mV.

F. REFERENCES

1. Swarts, J.C.; Langner, E.H.G.; Krokeide-Hove, N.; Cook, M.J. Synthesis and Electrochemical Characterisation of Some Long Chain 1,4,8,11,15,18,22,25-Octa-Alkylated Metal-Free and Zinc Phthalocyanines Possessing Discotic Liquid Crystalline Properties. *J. Mater. Chem.* **2001**, *11*, 434–443, doi:10.1039/b006123i.
2. Sharman, W.M.; Van Lier, J.E. Synthesis and Photodynamic Activity of Novel Asymmetrically Substituted Fluorinated Phthalocyanines. *Bioconjug. Chem.* **2005**, *16*, 1166–1175, doi:10.1021/bc0500241.
3. Terekhov, D.S.; Nolan, K.J.M.; McArthur, C.R.; Leznoff, C.C. Synthesis of 2,3,9,10,16,17,23,24-Octaalkynylphthalocyanines and the Effects of Concentration and Temperature on Their 1H NMR Spectra. *J. Org. Chem.* **1996**, *61*, 3034–3040, doi:10.1021/jo9521662.
